# Liver fibrosis prevalence and risk factors in patients with psoriasis: A systematic review and meta-analysis

**DOI:** 10.3389/fmed.2022.1068157

**Published:** 2022-12-15

**Authors:** Tanat Yongpisarn, Amornrut Namasondhi, Wimolsiri Iamsumang, Ploysyne Rattanakaemakorn, Poonkiat Suchonwanit

**Affiliations:** Division of Dermatology, Faculty of Medicine, Ramathibodi Hospital, Mahidol University, Bangkok, Thailand

**Keywords:** cirrhosis, hepatic fibrosis, hepatotoxicity, liver toxicity, NAFLD, NASH, non-alcoholic fatty liver disease, non-alcoholic steatohepatitis

## Abstract

**Background:**

Patients with psoriasis are more likely than matched controls in the general population to have advanced liver fibrosis; however, our understanding of these patients is limited. There is currently no systematic evaluation of the prevalence and risk factors of liver fibrosis in psoriasis patients.

**Objective:**

To evaluate the prevalence of psoriasis patients who are at high or low risk for advanced liver fibrosis and determine the risk factors for developing liver fibrosis.

**Methods:**

Electronic searches were conducted using the PubMed, Embase, Scopus, and Cochrane Library databases from the dates of their inception till May 2022, using the PubMed, Embase, Scopus, and Cochrane Library databases. Any observational study describing the prevalence and/or risk factors for liver fibrosis in patients with psoriasis was included.

**Results:**

Patients with psoriasis at high risk for advanced liver fibrosis had a pooled prevalence of 9.66% [95% confidence interval (CI): 6.92–12.75%, *I*^2^ = 76.34%], whereas patients at low risk for advanced liver fibrosis had a pooled prevalence of 77.79% (95% CI: 73.23–82.05%, *I*^2^ = 85.72%). Studies that recruited methotrexate (MTX)-naïve patients found a lower prevalence of advanced liver fibrosis (4.44, 95% CI: 1.17–9.22%, *I*^2^ = 59.34%) than those that recruited MTX-user cohorts (12.25, 95% CI: 6.02–20.08%, *I*^2^ = 82.34%). Age, sex, BMI, PASI score, psoriasis duration, MTX cumulative dose, and the prevalence of obesity, MTX users, diabetes mellitus, hypertension, dyslipidemia, and metabolic syndrome were not identified as sources of heterogeneity by meta-regression analysis. The pooled odds ratios for age >50 years, BMI > 30, diabetes mellitus, hypertension, dyslipidemia, and metabolic syndrome were 2.20 (95% CI: 1.42–3.40, *I*^2^ = 0%), 3.67 (95% CI: 2.37–5.68, *I*^2^ = 48.8%), 6.23 (95% CI: 4.39–8.84, *I*^2^ = 42.4%), 2.82 (95% CI: 1.68–4.74, *I*^2^ = 0%), 3.08 (95% CI: 1.90–4.98, *I*^2^ = 0%), and 5.98 (95% CI: 3.63–9.83, *I*^2^ = 17%), respectively.

**Conclusion:**

Approximately 10% of the population with psoriasis is at high risk for advanced liver fibrosis, while 78% are at low risk. Patients over the age of 50 with obesity, diabetes, hypertension, dyslipidemia, and/or metabolic syndrome have an increased risk of developing liver fibrosis, necessitating monitoring.

**Systematic review registration:**

[https://www.crd.york.ac.uk/prospero/display_record.php?ID=CRD42022303886], identifier [CRD42022303886].

## Introduction

Liver disease is a leading cause of mortality and morbidity worldwide (1). When liver fibrosis reaches an advanced stage, patients are at increased risk of developing hepatocellular carcinoma, hepatic decompensation, and liver-related mortality (1). Patients with psoriasis are more likely to have advanced liver fibrosis than matched controls in the general population (2). They are predisposed to liver fibrosis for a variety of reasons, one of which is non-alcoholic fatty liver disease (NAFLD), a prevalent liver disease affecting approximately 25% of the general population (3). NAFLD has been found to be strongly associated with psoriasis in previous meta-analyses (4–6).

Recent scientific breakthroughs have significantly advanced our understanding of psoriasis pathophysiology, resulting in the development of targeted biologic therapies such as anti-TNF, IL-12/23 inhibitors, IL-17 inhibitors, and IL-23 inhibitors. Furthermore, the efficacy of biologic treatments for psoriasis has been validated by numerous real-world studies (7–9), which is significant because, in a real-life situation, some patients are not typically included in clinical trials, such as those with multiple comorbidities, elderly patients, erythrodermic or pustular psoriasis, and previous history of biologic treatment failure. Moreover, paradoxical adverse effects, such as the development of hidradenitis suppurativa and vitiligo, have been linked to biologics in psoriasis patients and require to be monitored (10, 11).

Methotrexate (MTX) is among the most frequently prescribed non-biologic medications for psoriasis. In addition to its standard indications, such as psoriatic arthritis, MTX can be prescribed in conjunction with biologic therapy to minimize the occurrence of anti-drug antibodies (12). The extent of the association between MTX and liver fibrosis in psoriasis patients is disputed. A histology-based study discovered a similarity between the histopathologic features of MTX-induced liver toxicity and non-alcoholic steatohepatitis (NASH), a severe form of NAFLD, implying that MTX may exacerbate pre-existing NASH (13). As a result, psoriasis patients who have NASH risk factors such as diabetes or obesity are also classified as having a high risk of hepatotoxicity by psoriasis guidelines (14, 15), as they can develop liver fibrosis as a result of MTX toxicity at a lower cumulative dose.

Screening for liver fibrosis is critical because it identifies patients at risk, allowing elimination of hepatotoxic risk factors from those individuals proactively (such as by switching off hepatotoxic medications). Previous psoriasis guidelines recommend monitoring hepatotoxicity with routine blood sampling and liver biopsy (14, 15). However, a liver biopsy may be deemed excessively invasive, and many studies stated that routine liver enzyme tests are not sensitive enough to detect advanced liver fibrosis (16).

Recent research has demonstrated that non-invasive tests (NITs) are extremely beneficial in clinical practice, as they can reliably rule out the presence of advanced fibrosis in NAFLD patients (17). Patients classified as having a high risk of advanced fibrosis may be referred for additional testing, while those classified as having a low risk of advanced fibrosis may be offered lifestyle modifications and annual re-evaluation (17). NIT has been incorporated into the most recent AAD guidelines for MTX hepatotoxicity screening (18). NIT is recommended for a baseline evaluation of liver fibrosis; if NIT reveals a low risk of liver fibrosis, MTX can be initiated, and annual evaluations are recommended (18).

Liver stiffness measurement (LSM) by transient elastography (TE) is the most extensively used and verified non-invasive technique to date, and is frequently referred to as the NIT of choice by many. However, the tests to be used (serum biomarkers or imaging-based techniques) should be determined by local availability and usage context. For example, because TE performs poorly in obese individuals, alternative techniques such as magnetic resonance elastography or point shear wave elastography may be considered depending on local availability. While in resource-limited settings, liver fibrosis scores calculated from simple laboratory values such as the Fibrosis-4 index (FIB-4) may be used to identify patients who require additional testing such as TE and liver biopsy (19).

The prevalence of advanced liver fibrosis in patients with psoriasis is not well established, which contributes to a lack of awareness regarding the risk of advanced liver fibrosis in psoriasis patients. To the best of our knowledge, no meta-analysis has been conducted to determine the prevalence or risk factors for liver fibrosis in patients with psoriasis. The objectives of this meta-analysis were to determine the prevalence of patients with psoriasis who were at a high or low risk of having advanced liver fibrosis, as well as to determine the risk factors for developing liver fibrosis. Our research, we believe, will inform future clinical decision-making regarding risk assessment, screening, and treatment of psoriasis patients in daily practice, thereby promoting more tailored care and improving patient outcomes.

## Materials and methods

### Study design

The protocol was registered in PROSPERO (International Prospective Register of Systematic Reviews; no. CRD42022303886). The systematic review followed the Preferred Reporting Items for Systematic Reviews and Meta-analyses guidelines (20) (Supplementary document). Electronic searches were conducted from database’s inception to May 2022, using the PubMed, Embase, Scopus, and Cochrane Library databases. Using keywords and a controlled vocabulary, the search strategy was designed to retrieve all studies on psoriasis and NITs for liver fibrosis. There were no restrictions on the language or publication period of the searches. Conference abstracts were excluded. Supplementary Table 1 provides details about the search strategy.

### Study selection

Each article was reviewed independently by two reviewers (TY and AN), both at the title/abstract and full-text levels. Disagreements between the two reviewers regarding the studies’ eligibility were resolved *via* discussion with a third reviewer (WI). Any observational study describing the prevalence and/or risk factors for liver fibrosis in patients with psoriasis or containing sufficient data to calculate the respective prevalence or odds ratio was included. We excluded studies in which more than 10% of patients had a known cause of liver fibrosis (except NAFLD), such as chronic viral hepatitis, or consumed excessive amounts of alcohol (more than 20 g/day). We also excluded studies on special populations such as pregnant patients, duplicate studies from the same cohort, and studies with a small sample size of fewer than 10.

### Data extraction

Data were extracted from the included studies using a standardized format. The following data were collected: study type, study characteristics (primary author, country, publication year), patient characteristics [number of psoriasis patients, age, female percentage, weight, BMI, severity of psoriasis (PASI), age of onset, disease duration, type of psoriasis, presence of joint involvement, systemic treatment with duration and accumulative dosage, relevant laboratory data, alcohol intake, comorbidity], investigations [tests for liver fibrosis with associated cutoff(s), liver biopsy], and outcomes (prevalence of liver fibrosis detected, sensitivity and specificity, risk ratios of associated factors for significant fibrosis). Corresponding investigators were contacted *via* email if there was missing data. Two independent reviewers (TY and AN) extracted data, and discrepancies were resolved with the assistance of a third reviewer (WI).

### Quality assessment

TY and AN independently assessed the quality of cohort and case-control studies using the adapted version of the Newcastle-Ottawa Scale (NOS) (21). The original NOS is a scoring tool comprised of seven items with nine scores that assesses how well the investigators selected their participants (score ranges from 0 to 4), the comparability of their results (score ranges from 0 to 2), and the applicability of the outcomes (score ranges from 0 to 3). We assigned up to one point for the sample size element of the selection score since smaller studies are prone to sampling bias; hence we had to lower the outcome score from 3 to 2. The higher the score, the higher the study’s quality and the lower the likelihood of bias. Therefore, we classified studies as having high quality if they received a total score of 7 or more, fair quality if they received a score of 4–6, and low quality if they received a score of 4. Any discrepancies between reviewers regarding the risk of bias in specific studies were resolved through discussion with a third reviewer (PR).

### Statistical analysis

Primary analysis assessed the prevalence of psoriasis patients with low and high risk of advanced liver fibrosis. The odds ratios for variables associated with liver fibrosis were also pooled using the inverse variance method. The “metaprop” and “metan” commands were used in Stata to summarize prevalence and odds ratios, respectively (22). As significant heterogeneity across studies was expected, the DerSimonian–Laird random-effects model was used. Heterogeneity between studies was estimated using Higgins’ and Thompson’s *I*^2^-statistics derived from Cochran’s *Q*-test, with an *I*^2^ value > 50% representing substantial heterogeneity (23).

The prevalence analyses were based on previously established cutoffs for low risk (LSM < 8 kPa, FIB-4 < 1.3, NFS < −1.455, FibroTest/FibroSure < 0.3) and high risk (LSM ≥ 10 kPa, FIB-4 > 3.25, NFS > 0.672, ELF > 9.8, FibroTest/FibroSure > 0.7) of advanced liver fibrosis (17, 24, 25). When different cutoffs were utilized in the included studies, we selected those closest to the established cutoffs and categorized them accordingly. Because TE is the current test of choice, it was chosen to represent the cohort’s prevalence in studies that included multiple tests. When multiple NITs were used to determine the prevalence of a cohort and none of the NITs were TE, the NIT with the highest performance in detecting advanced fibrosis and cirrhosis was selected to represent the cohort’s prevalence (17, 26). We could not locate any study demonstrating that an abnormal level of procollagen III amino-terminal peptide (PIIINP) is associated with an increased risk of advanced liver fibrosis; instead, all such studies were conducted regardless of fibrosis stage, and thus PIIINP studies were excluded from the quantitative analysis. Additionally, studies that lacked data specifically on psoriasis patients were excluded from the quantitative analysis.

To compare the prevalence estimated between groups and investigate the source of heterogeneity, subgroup analyses by the geography of research origin, type of NIT, and percentage of MTX users were conducted. We also conducted univariable meta-regression analyses on variables with at least ten observations to determine the effect of specific moderators on the prevalence of liver fibrosis across studies (e.g., age, sex, and BMI). Deeks funnel plots of the outcomes were created to assess for publication bias. Due to the possibility of bias revealed by funnel plots, the Egger linear regression test was used. All statistical analyses were conducted using STATA 16.0 (StataCorp LLC, College Station, TX, USA).

## Results

### Study characteristics

**FIGURE 1:**
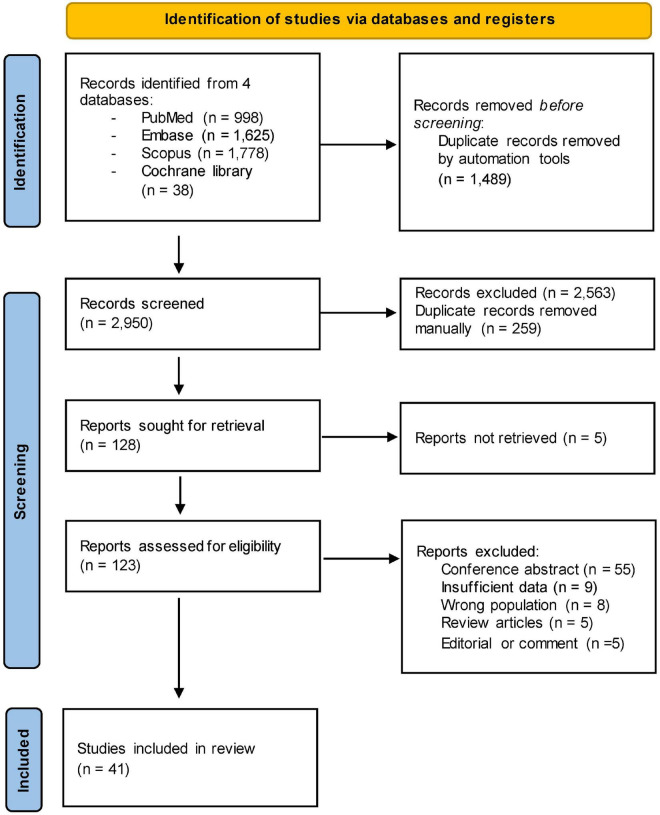
Preferred reporting items for systematic reviews and meta-analyses (PRISMA) flow diagram of search strategy and included studies.

After removing duplicates, 2,619 references were screened by title/abstract. At the full-text stage, 128 full articles met our predefined selection criteria and were sought, 123 were retrieved, and we further excluded 82 references for the following reasons: conference abstract (*n* = 55), insufficient data (*n* = 9), wrong population (*n* = 8), review articles (*n* = 5), and editorial or comment (*n* = 5) (Figure 1). Forty-one studies, enrolling a total of 3,868 patients with psoriasis between 1988 and 2022, were included in the review (Table 1) (Supplementary Table 2).

**TABLE 1:** Characteristics of included studies.

References	Country	Number of patients, age (years), female	Prevalence of patients with high and low risk of advanced liver fibrosis NIT and cutoff
Lee et al. (27)	Malaysia	**MTX group:** 61 patients, age 52.98 ± 15.5, female 29 (47.5%)	• **Low risk:** LSM < 6.5 kPa = 39 (63.9%), FIB-4 < 1.45 = 48 (78.7%), APRI < 0.7 = 54 (88.5%) • **High risk:** LSM > 11.5 kPa = 7 (11.5%), FIB-4 > 3.25 = 2 (3.3%), APRI > 1.0 = 5 (8.2%)
		**MTX-naïve group**: 56 patients, age 52.2 ± 15.1, female 24 (42.9%)	• **Low risk:** LSM < 6.5 kPa = 45 (80.4%), FIB-4 < 1.45 = 46 (82.1%), APRI < 0.7 = 49 (87.5%)****• **High risk:** LSM > 11.5 kPa = 4 (7.1%), FIB-4 > 3.25 = 2 (3.6%), APRI > 1.0 = 6 (10.7%)
Mahajan et al. (30)	India	61 patients, age 47.5 ± 13.8, female 18 (29.5%)	• **Low risk:** LSM < 7.6 kPa = 61 (100%)
Takamura et al. (28)	Japan	65 patients, age 46 (range 40–54.5), female 16 (24.6%)	• **Low risk:** NFS < −1.455 = 75.4%, FIB-4 < 1.3 = 76.9%
Rattanakaemakorn et al. (31)	Thailand	132 patients, age 52, female 68 (51.5%)	• **Low risk:** LSM < 8 kPa = 123 (93.2%)****• **High risk:** LSM ≥ 10 kPa = 7 (5.3%)
Belinchoìn-Romero et al. (32)	Spain	91 patients (87 with valid TE), age 53 (IQR 45.5–61.5), female 32 (35.2%)	• **Low risk:** LSM < 7.8 kPa = 72 (82.8%)
Brunner et al. (33)	Hungary	52 patients, age 54.0 ± 13.4, female 26 (50%)	• **Low risk:** LSM < 8.2 kPa = 72 (82.8%) • **High risk:** LSM > 9.7 kPa = 14 (26.9%)
Cervoni et al. (34)	France	66 patients (49 TE, 63 Forns index, 65 APRI, 65 FIB-4, 64 FPI, 64 Hepascore, 64 NFS, 64 Fibrometer, 61 FibroTest), age 54 ± 2, female 39%	• **Low risk:** LSM < 7.1 kPa = 90.5%, FibroTest < 0.49 = 88.6%, Hepascore < 0.5 = 92.2%, Forns Index < 6.9 = 90.5%, FPI < 0.8 = 87.5%, FibroMeter < 0.49 = 78.1% • **High risk:** FIB-4 > 3.25 = 4.6%, NFS > 0.676 = 6.3%, APRI > 1.5 = 3.1%
Yim et al. (19)	Spain	39 patients, age 49.8, female 24 (61.5%)	• **Low risk:** FIB-4 < 1.45 = 26 (66.7%)****• **High risk:** FIB-4 > 3.25 = 1 (2.6%)
Rivera et al. (35)	Spain	457 patients (280 NFS, 392 FIB-4), age 53.3 ± 14.0, female 199 (43.5%)	• **Low risk:** FIB-4 < 1.3 = 73.8%, NFS < −1.455 = 62.8%
Koch, (36)	New Zealand	66 patients, age 51.2 ± 14.0, female 34 (51.5%)	• **Low risk:** LSM < 7.1 kPa = 37 (56.1%) • **High risk:** LSM > 9 kPa = 23 (34.8%)****• PIIINP > 4.2 μg/L = 29 (43.9%)
Mahajan et al. (29)	India	134 patients, age 44.13 ± 13.86, female 40 (30.3%)	• **Low risk:** LSM < 7 kPa = 101 (75.4%) • **High risk:** LSM ≥ 9 kPa = 16 (6.7%)
Magdaleno-Tapial et al. (37)	Spain	71 patients, age 46.7 ± 14 years, female 24 (33.8%)	• **Low risk:** LSM < 7.7 kPa = 61 (85.9%) • **High risk:** LSM ≥ 9.5 kPa = 6 (8.5%)
Kumar and Ganapathi, (38)	India	102 patients, age 42.12, females 61 (59.8%)	• **Low risk:** LSM < 7.5 kPa = 83 (81.3%) • **High risk:** LSM ≥ 10 kPa = 8 (7.8%)
Neema, (39)	India	82 patients, age 47.04 ± 12.45, female 20 (24.4%)	• **Low risk:** LSM < 7 kPa = 59 (72.0%)
Ortolan et al. (40)	Italy	**PsA group:** 43 patients, age 60.2 ± 8.4, female 11 (25.6%)	• **Low risk:** LSM < 7 kPa = 69%
		**without PsA group:** 33 patients, age 54.5 ± 19.6, female 12 (36.4%)	• **Low risk:** LSM < 7 kPa = 72%
Ben Lagha et al. (41)	Tunisia	88 patients, age 45.6 ± 14.3, female 48 (42.9%)	• **Low risk:** LSM < 7 kPa = 71 (80.7%) • **High risk:** LSM > 9.5 kPa = 5 (5.7%)
Maybury et al. (42)	United Kingdom	400 patients (333 TE), age 49.5 ± 13, female 108 (27.2%)	• **Low risk:** LSM < 7 kPa = 265 (79.6%) • **High risk:** LSM 8.7 kPa = 47 (14.1%)
Van den Reek et al. (55)*	Netherlands	**Elevated PIIINP:** 41 patients, age 55.9 ± 16.5, female 20 (48.8%)	• Elevated PIIINP = 41 (22.4%)
		**No elevated PIIINP:** 142 patients, age 55.9 ± 16.5, female 20 (48.8%)	
Van der Voort et al. (54)	Netherlands	**PsA group:** 151 patients, age 52.8 ± 11.7 years, female 70 (46.3%)	• **High risk:** ELF > 9.8 = 20 (13.2%) • PIIINP > 12.2 μg/L = 7 (6%)****⋅ PIIINP > 15.3 μg/L = 6 (5.2%)
		**Without PsA group:** 119 patients, age 49.8 ± 14.3, female 45 (37.8%)	• **High risk:** ELF > 9.8 = 25 (21%)****• PIIINP > 12.2 μg/L = 9 (6%)****• PIIINP > 15.3 μg/L = 2 (1.3%)
Bauer et al. (43)	United States	107 patients (69 FibroSure), age 83.3 ± 13.5, female 57 (53.2%)	• **Low risk:** FibroSure < 0.21 = 50 (71.5%)
Talme et al. (44)	Sweden	**Biologic group:** 32 patients, age 48 (range 18–76), female 6 (18.8%)	• **Low risk:** LSM < 6.5 kPa = 20 (62.5%) • **High risk:** LSM > 11.5 kPa = 1 (3.1%)
		**MTX duration > 24 months group**: 122 patients, age 60 (range 22–82), female 52 (41.9%)	• **Low risk:** LSM < 6.5 kPa = 76 (62.3%)****• **High risk:** LSM > 11.5 kPa = 11 (9%)
		**MTX duration ≤ 24 months group**: 47 patients, age 50 (range 20–76), female 17 (34.7%)	• **Low risk:** LSM < 6.5 kPa = 32 (68.1%)****• **High risk:** LSM > 11.5 kPa = 3 (6.4%)
Rongngern et al. (45)	Thailand	41 patients, age 51.2 ± 11.6, female 17 (41.5%)	• **Low risk:** LSM < 7.1 kPa = 31 (75.6%) • **High risk:** LSM ≥ 10 kPa = 3 (7.3%)
Pongpit et al. (46)	Thailand	165 patients, age 49.2 ± 14, female 90 (54.5%)	• **Low risk:** LSM < 7 kPa = 147 (89.1%) • **High risk:** LSM > 9.5 kPa = 11 (6.7%)
Gisondi et al. (47)	Italy	124 patients (55 NFS), age 55 ± 12, female 55 (44%)	• **Low risk:** NFS < −1.455 = 30 (54.5%) • **High risk:** NFS > 0.676 = 4 (7.3%)
Van der Voort et al. (53)	Netherlands	74 patients, age 71.2 ± 6.5 years, female 33 (44.6%)	• **High risk:** LSM > 9.5 kPa = 6 (8.1%)
Martyn-Simmons et al. (65)*	United Kingdom	27 patients, age 56 ± 2.7 years, female 9 (33%)	NR
Lynch et al. (48)	Ireland	77 patients (50 TE, 70 FibroTest, 51 PIIINP), age 51 (range 22–85), female 36 (46.8%)	• **Low risk:** LSM < 7 kPa = 41 (82%), FibroTest < 0.31 = 59 (84.3%) • Serial PIIINP (1 year before TE) > 4.2 μg/L = 9/51 (17.6%) • Serial PIIINP (1 year before FibroTest) > 4.2 μg/L = 3/34 (8.8%)
Bray et al. (49)	United Kingdom	21 patients (10 TE), age 59 (range 41–83), female 9 (42.9%)	• **Low risk:** LSM < 8 kPa = 6 (60%) • **High risk:** LSM ≥ 10 kPa = 3 (30%)
Madanagobalane and Anandan, (50)	India	58 patients, age 46.9 ± 1.15, female 12 (20.7%)	• **Low risk:** FibroTest stage < F2–F3 = 94.9%
Seitz et al. (51)	Switzerland	**TNF-naïve group**: 20 patients, age 51.9 ± 14.1, female 6 (30.0%)	• **Low risk:** LSM < 8 kPa = 14 (70%)
		**TNF group:** 23 patients, age 51.3 ± 10.9, female 7 (30.4%)	• **Low risk:** LSM < 8 kPa = 22 (95.7%)
Laharie et al. (52)	France	111 patients, age 56.2 ± 12.2, female 30 (27%)	• **Low risk:** LSM < 7.9 kPa = 99 (89.2%)
Lindsay and Gough, (56)*	United Kingdom	48 patients, age 54.4 ± 11, female NR	• Elevated PIIINP = 16 (33.3%)
Berends et al. (66)*	Netherlands	24 patients, age 55 (range 34–73), female 13 (54.2%)	NR
Khan et al. (64)*	United Kingdom	15 patients, age 56.4 ± 12.8, female 7 (46.7%)	NR
Zachariae et al. (57)*	Denmark	70 patients, age NR, female 31 (44.3%)	• PIIINP > 4.2 μg/L = 6 (8.6%)
Zachariae et al. (59)*	Denmark	11 patients, age NR, female NR	• PIIINP > 4.2 μg/L = 0
Boffa et al. (60)*	United Kingdom	87 patients, age NR, female NR	• PIIINP > 4.2 μg/L = 41 (47.1%)
Oogarah et al. (63)*	United Kingdom	22 patients, age 42.6 (22–72), female 8 (36.4%)	• Elevated PIIINP (first assay) = 6/11 (54.5%) • Elevated PIIINP (second assay) = 4/22 (18.2%)
Zachariae et al. (58)*	Denmark	170 patients, age NR, female NR	• PIIINP > 4.3 μg/L = 24 (21.8%)
Mitchell et al. (61)*	United Kingdom	51 patients, age 47 (range 22–69), female NR	• PIIINP > 11.8 μg/L = 35 (68.6%)
Risteli et al. (62)*	Denmark	24 patients, age 50 (range 32–75), female 10 (41.7)	• PIIINP > 4.2 μg/L = 8 (33.3%)

APRI, aspartate aminotransferase to platelet ratio index; ELF, enhanced liver fibrosis; FIB-4, fibrosis-4 index; FPI, fibrosis probability index; LSM, liver stiffness measurement; MTX, methotrexate; NFS, non-alcoholic fatty liver disease fibrosis score; NR, not reported; PsA, psoriasis arthritis; PIIINP, procollagen III amino-terminal peptide; TE, transient elastography; TNF, tumor necrosis factor alpha blockers; UK, United Kingdom; USA, United States. *These studies are not included in the quantitative analysis.

We included thirty-seven studies in the quantitative analysis (19, 27–62), and twelve studies were only used qualitatively (55–66). Nine types of NIT were included: twenty-two TE (27, 29–34, 36–42, 44–46, 48, 49, 51–53, 66) (*n* = 2222), thirteen PIIINP (36, 54–65) (*n* = 947), six FIB-4 (19, 27, 28, 32, 34, 35) (*n* = 689), five NAFLD fibrosis score (NFS) (28, 32, 34, 35, 47) (*n* = 477), four FibroTest/FibroSure (34, 43, 50, 66) (*n* = 285), two AST to Platelet Ratio Index (APRI) (27, 34) (*n* = 180), two Enhanced Liver Fibrosis score (ELF) (54, 65) (*n* = 297), one Forns Index (34) (*n* = 63), one FibroMeter (34) (*n* = 64), one Hepascore (34) (*n* = 64), and one Fibrosis Probability Index (34) (*n* = 64) studies. Formulae of NITs mentioned in this review are shown in Supplementary Table 3.

### Prevalence of high risk of advanced liver fibrosis

**FIGURE 2:**
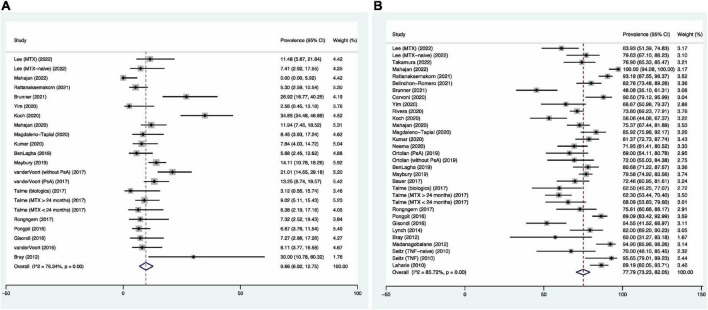
Forest plots for pooled prevalence of psoriasis patients with high risk **(A)** and low risk **(B)** of advanced liver fibrosis.

The pooled prevalence of 9.66% [95% confidence interval (CI): 6.92–12.75%, *I*^2^ = 76.34%, Figure 2A] was found for psoriasis patients with a high risk of having advanced liver fibrosis. Studies conducted in Asian countries (6.64, 95% CI: 3.92–9.94%, *I*^2^ = 59.08%) were found to have lower prevalence, compared to European countries (10.99, 95% CI: 7.66–14.79%, *I*^2^ = 64.45%). Fifteen of the 18 studies that used NIT with a high-risk cutoff for advanced liver fibrosis performed TE as their NIT, and the prevalence was 9.36% (95% CI: 6.35–12.82%, *I*^2^ = 76.34%). Subgroup analyses based on other NITs were not performed due to a limited number of studies. A lower prevalence (4.44, 95% CI: 1.17–9.22%, *I*^2^ = 59.34%) was also found among studies that recruited MTX-naïve patients, compared to MTX-user cohorts (12.25, 95% CI: 6.02–20.08%, *I*^2^ = 82.34%) (67) (Supplementary Figure 1).

### Prevalence of low risk of advanced liver fibrosis

The pooled prevalence of 77.79% (95% CI: 73.23–82.05%, *I*^2^ = 85.72%, Figure 2B) was found for psoriasis patients with a low risk of advanced liver fibrosis. Studies conducted in Europe (74.61, 95% CI: 68.88–79.96%, *I*^2^ = 80.49%) were found to have lower prevalence than studies originated in Asia (84.02, 95% CI: 76.48–90.39%, *I*^2^ = 88.04%). Twenty-one of the 27 studies that used NIT with a low-risk cutoff for advanced liver fibrosis used TE as their NIT, and the prevalence was found to be 78.51% (95% CI: 78.51–83.36%, *I*^2^ = 85.97%). Whereas, in the studies that used FIB-4 (3 studies) and FibroTest/FibroSure (2 studies) as their NIT, the prevalences were 73.88 and 84.39%, respectively. Similar prevalence was found for MTX-naïve and MTX-user cohorts, with pooled prevalence of 79.98% (95% CI: 65.79–91.23%, *I*^2^ = 90.89%) and 71.95% (95% CI: 64.70–78.69%, *I*^2^ = 83.54%) (Supplementary Figure 2).

### Meta-regression

Meta-regression analysis did not identify age, BMI, PASI score, psoriasis duration, MTX cumulative dose, and the proportion of females, obesity, MTX user, diabetes mellitus, hypertension, dyslipidemia, and metabolic syndrome as sources of heterogeneity. Supplementary Table 4 provides a summary of the meta-regression analysis.

### Factors associated with liver fibrosis

The pooled odds ratios of 2.20 (95% CI: 1.42–3.40, *I*^2^ = 0%), 3.67 (95% CI: 2.37–5.68, *I*^2^ = 48.8%), 6.23 (95% CI: 4.39–8.84, *I*^2^ = 42.4%), 2.82 (95% CI: 1.68–4.74, *I*^2^ = 0%), 3.08 (95% CI: 1.90–4.98, *I*^2^ = 0%), and 5.98 (95% CI: 3.63–9.83, *I*^2^ = 17%) were found for age > 50 years, BMI > 30, diabetes mellitus, hypertension, dyslipidemia, and metabolic syndrome, respectively. Pooled odds ratios of 1.10 (95% CI: 0.87–1.39, *I*^2^ = 3.2%), 1.67 (95% CI: 0.94–2.95, *I*^2^ = 37%), 1.30 (95% CI: 0.82–2.06, *I*^2^ = 30.6%), and 1.58 (95% CI: 0.91–2.75, *I*^2^ = 0%) were found for male, PASI > 10, psoriatic arthritis, and cumulative MTX dose > 1500 mg, respectively. Supplementary Figure 3 depicts the forest plots, and Table 2 summarizes the details.

**TABLE 2:** Summary of pooled odds ratios of variables associated with significant liver fibrosis.

Variables	Pooled OR (95% CI)	*I* ^2^	References	Number of patients
Age	2.20 (1.42–3.40)	0%	(31, 38, 44, 45)	344
Male	1.10 (0.87–1.39)	3.2%	(27, 29, 31, 35, 36, 38, 39, 41, 42, 44–46)	1,851
PASI > 10	1.67 (0.94–2.95)	37%	(27, 31, 41, 46)	500
Psoriatic arthritis	1.30 (0.82–2.06)	30.6%	(27, 35, 36, 46)	738
Cumulative MTX dose >1500 mg	1.58 (0.91–2.75)	0%	(29, 39, 45, 46)	422
BMI > 30	3.67 (2.37–5.68)	48.8%	(27, 31, 36, 41, 44)	602
Diabetes mellitus	6.23 (4.39–8.84)	42.4%	(27, 31, 35, 36, 41, 44–46)	1,200
Hypertension	2.82 (1.68–4.74)	0%	(27, 41, 45, 46)	409
Dyslipidemia	3.08 (1.90–4.98)	0%	(27, 31, 41, 45, 46)	541
Metabolic syndrome	5.98 (3.63–9.83)	17%	(35, 39, 41, 45, 46)	768

BMI, body mass index; MTX, methotrexate; OR, odds ratio; PASI, psoriasis area severity index.

### Quality assessment and publication bias

**FIGURE 3:**
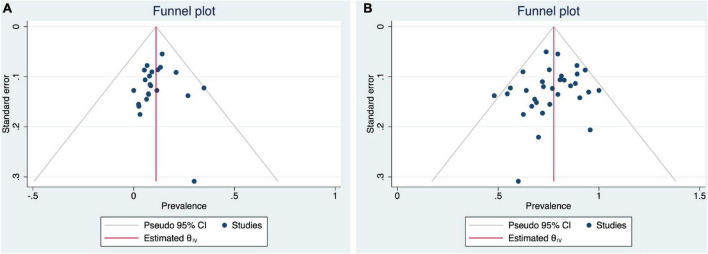
Funnel plots for pooled prevalence of psoriasis patients with **(A)** high risk and **(B)** low risk of advanced liver fibrosis analyses.

Supplementary Table 5 summarizes the quality assessment scores for the included studies. The mean quality assessment score was 8.1 (range: 4–9), with 34 high-quality studies and seven of moderate quality. Publication bias was assessed through funnel plots, which were found to be slightly asymmetric (Figure 3), but Egger’s tests (*p* = 0.86 and 0.33) indicate no publication bias.

## Discussion

In this systematic review and meta-analysis, we discover that 9.66% of people with psoriasis are at high risk of having advanced liver fibrosis, necessitating further investigation and management. While 77.79% of the population is considered low risk, the remaining 22.21% requires further testing. Subgroup analysis revealed a lower prevalence of advanced liver fibrosis in Asian studies (6.64%), compared to European studies (10.99%). It is highly improbable to be the result of NAFLD alone, as a recent meta-analysis (68) discovered comparable prevalence of NAFLD in Asia (30.5%) and Europe (30.9%). Further investigation of the factors that contribute to geographic disparity may provide additional insights on risk factors for advanced liver fibrosis in patients with psoriasis.

We are able to identify significant risk factors for liver fibrosis, including advanced age, diabetes mellitus, hypertension, dyslipidemia, and metabolic syndrome. On the other hand, disease-related factors such as severe psoriasis and psoriatic arthritis were not found to have a statistically significant association with liver fibrosis. Our findings are consistent with previous meta-analyses (4–6) which found that NAFLD was associated with the development of liver fibrosis in patients with psoriasis. Furthermore, a previous study by Ortolan et al. also reported that insulin resistance was the main determinant of liver fibrosis, and psoriatic arthritis, a disease-related inflammation, was not a significant predictor (40). As the evidence for MTX-induced liver fibrosis is anecdotal and based on poorly controlled data, metabolic syndrome and its risk factors, such as insulin resistance, could be considered a more significant risk factor for liver fibrosis than MTX use (69–75).

Since the introduction of NITs, routine liver biopsy on psoriasis patients has become unethical. The most recent joint AAD-NPF guideline does not recommend a baseline liver biopsy, regardless of the presence of risk factors for liver fibrosis (18). Liver biopsies are now reserved for patients who have been classified non-invasively as having a high risk of advanced liver fibrosis and who have been advised to undergo the procedure by a gastrointestinal specialist (18).

There is limited evidence regarding the performance of different NITs in detecting advanced liver fibrosis in the psoriasis population, which may contribute to their underutilization. Only three studies using TE reported sensitivity and specificity values ranging from 50% (45, 66) to 100% (49) and 67 (49) to 88% (66), respectively, for detecting significant liver fibrosis in psoriasis patients. Previous meta-analysis (16) reported a pooled sensitivity and specificity for TE of 60 and 80%, respectively; however, the number of patients included was limited (two studies, *n* = 34).

In the past, PIIINP was used to detect liver fibrosis in patients with psoriasis taking MTX and was found to have a pooled sensitivity and specificity of 74 and 77% (16), respectively, for any stage of liver fibrosis. In addition to not being widely available in many countries, including the United States (18), it is well-established that PIIINP has other significant limitations, including the fact that it only measures active fibrogenesis and may be falsely elevated in inflammatory conditions such as arthritis (76–82). We observed that the majority of PIIINP studies were small, old, and received lower scores by NOS. Since the introduction of other NITs, the number of published PIIINP studies has decreased. In the last decade, only four studies (36, 48, 54, 55) involving PIIINP have been conducted, and none of them have recommended PIIINP over other tests. More studies on PIIINP are needed to justify its usage, particularly on how a particular level of PIIINP would be associated with high risk of advanced liver fibrosis. In the meantime, we concur with Patel that other NITs, such as FIB-4, may be preferred because they are more consistent with current recommendations (83).

There is an urgent need for a safe, non-invasive, and efficient screening test to aid in the diagnosis or exclusion of advanced liver fibrosis in psoriasis patients. Many studies (19, 27, 34, 37) attempted to assess the risk of liver fibrosis without performing a biopsy; however, we felt that the efficacy of NITs had not been established in the psoriasis population. Future large-scale, prospective, histologically based studies comparing the performance of various tests for the detection of liver fibrosis are necessary to determine which tests are the most effective and safe non-invasive screening tools for liver fibrosis.

Methotrexate is one of the most frequently prescribed systemic medications for psoriasis patients due to its affordability and efficacy. In the past, the AAD recommended a routine liver biopsy for psoriasis patients who had received 3.5 to 4 g of MTX cumulative dosage due to the long-held belief that drug accumulation directly causes liver injury (84). In high-risk patients, it was recommended that liver biopsy be performed at a lower cumulative MTX dose of 1.0 to 1.5 g. The relationship between cumulative MTX dose and the risk of liver fibrosis in psoriasis patients remains controversial. A previous meta-analysis of histology-based studies found that MTX-induced liver toxicity is associated with total cumulative dose (85). In contrast, a recent systematic review of eight observational studies involving 429 psoriasis patients reported no clear association between cumulative MTX dose and liver fibrosis (69). Additionally, many observational studies (27, 33, 46, 48, 52, 66, 86) did not find any association between MTX cumulative dose and the risk of liver fibrosis. Lynch et al. discovered an association between MTX cumulative dose and abnormal FibroTest results, but not with TE (48). Bauer et al. discovered that a higher cumulative MTX dose was associated with a higher FibroSure score in women, but not in men (43). In our meta-analysis, we found that MTX-naive cohorts (4.44%) have a lower prevalence of patients at high risk for advanced liver fibrosis than MTX-using cohorts (12.25%). However, no statistically significant association between cumulative MTX doses greater than 1,500 mg and an increased risk of liver fibrosis was found. Additional research into the association between MTX use and liver fibrosis would aid in determining how strict a fibrosis screening protocol should be following MTX prescription.

The present results should be interpreted in the light of some limitations. First, there was substantial heterogeneity found in some of our analyses, specifically in the pooled prevalence analyses. Through multiple subgroup analyses and meta-regression, we investigated the source of the heterogeneity; however, we were unable to determine its source. Additionally, there are limitations to the fibrosis assessment. In contrast to previous reviews (69, 85) that included studies that assessed liver fibrosis through liver biopsies, we chose to examine the prevalence of patients at risk for liver fibrosis and the risk factors for liver fibrosis using NITs, as these are the method of choice when evaluating liver fibrosis for the first time in clinical practice.

## Conclusion

This meta-analysis sheds light on the burden of advanced liver fibrosis in psoriasis patients. We hope to inform practitioners and future researchers about the high prevalence of advanced liver fibrosis in psoriasis patients, as well as the critical need for liver fibrosis screening. Approximately 10% of the psoriasis population is at high risk of having advanced liver fibrosis, while only around 78% are at low risk. Patients over the age of 50 with comorbidities such as obesity, diabetes mellitus, hypertension, dyslipidemia, and/or metabolic syndrome are at an increased risk of developing liver fibrosis, necessitating surveillance. Further research is required to determine why the prevalence of patients at high risk for advanced liver fibrosis is higher in European countries, the performance of NITs for the detection of advanced liver fibrosis in patients with psoriasis, and the extent of association between MTX use, particularly its cumulative dose, and liver fibrosis.

## Data availability statement

The original contributions presented in this study are included in the article/Supplementary material, further inquiries can be directed to the corresponding author.

## Author contributions

WI, PR, and PS: conceptualization. PS, TY, and AN: methodology. PS and PR: validation. TY and WI: formal analysis. TY, AN, and WI: investigation. TY: data curation. TY, AN, WI, and PR: writing – original draft preparation. PS: writing – review and editing. All authors have read and agreed to the published version of the manuscript.
