# Supplementary material for: Liver fibrosis prevalence and risk factors in patients with psoriasis: A systematic review and meta-analysis
Source: Front Med (Lausanne). 2022 Dec 15;9:1068157. doi: 10.3389/fmed.2022.1068157 (PMC9797863; doi:10.3389/fmed.2022.1068157)
Supplement: Supplementary file 1 [file Data_Sheet_1.pdf]

Supplementary Figure 1: Forrest plots for pooled prevalence psoriasis of high risk of advanced liver fibrosis subgrouped based on (A) continent, (B) non-invasive tests, and (C) methotrexate user percentage

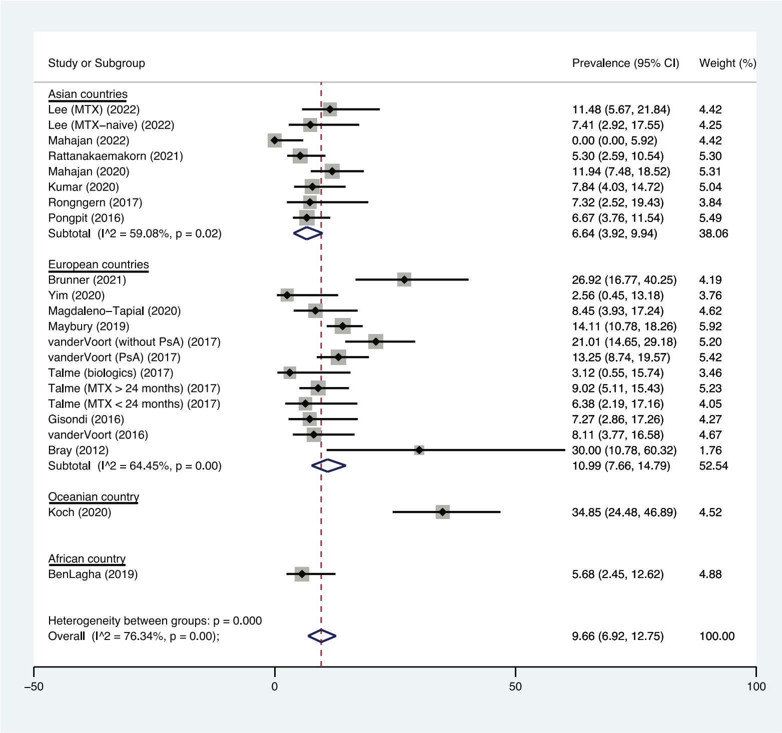

(A)

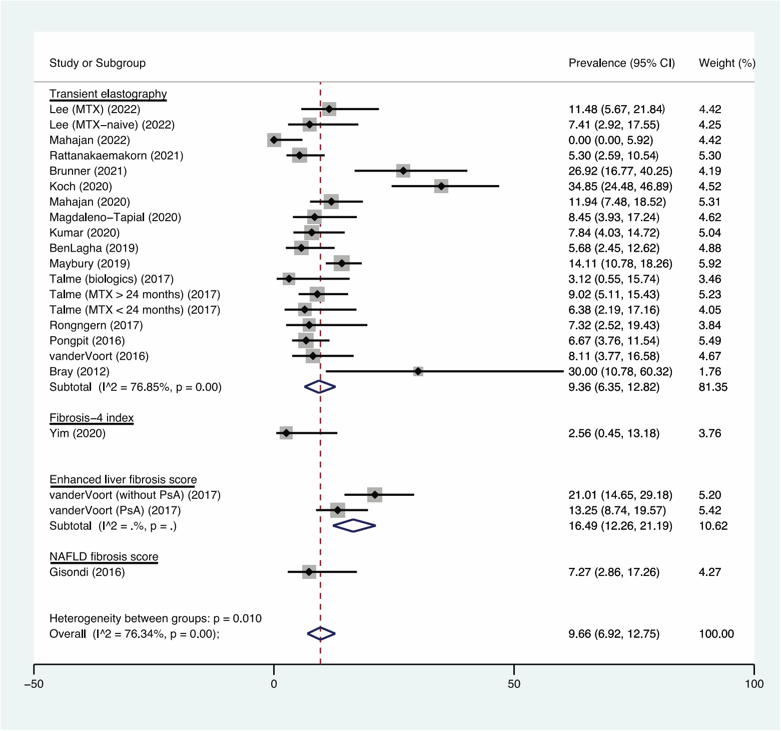

(B)

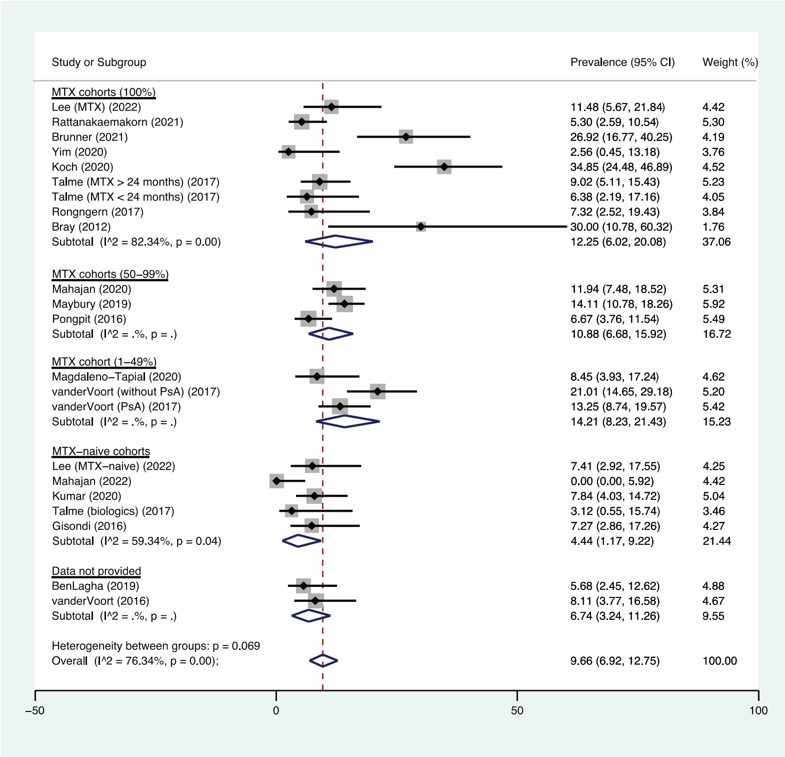
Study or Subgroup
Prevalence (95% CI)
Weight (%)
MTX cohorts (100%)
Lee (MTX) (2022) 11.48 (5.67, 21.84) 4.42
Rattanakaemakorn (2021) 5.30 (2.59, 10.54) 5.30
Brunner (2021) 26.92 (16.77, 40.25) 4.19
Yim (2020) 2.56 (0.45, 13.18) 3.76
Koch (2020) 34.85 (24.48, 46.89) 4.52
Talme (MTX > 24 months) (2017) 9.02 (5.11, 15.43) 5.23
Talme (MTX < 24 months) (2017) 6.38 (2.19, 17.16) 4.05
Rongngern (2017) 7.32 (2.52, 19.43) 3.84
Bray (2012) 30.00 (10.78, 60.32) 1.76
Subtotal (I^2 = 82.34%, p = 0.00) 12.25 (6.02, 20.08) 37.06
MTX cohorts (50–99%)
Mahajan (2020) 11.94 (7.48, 18.52) 5.31
Maybury (2019) 14.11 (10.78, 18.26) 5.92
Pongpit (2016) 6.67 (3.76, 11.54) 5.49
Subtotal (I^2 = .%, p = .) 10.88 (6.68, 15.92) 16.72
MTX cohort (1–49%)
Magdaleno–Tapial (2020) 8.45 (3.93, 17.24) 4.62
vanderVoort (without PsA) (2017) 21.01 (14.65, 29.18) 5.20
vanderVoort (PsA) (2017) 13.25 (8.74, 19.57) 5.42
Subtotal (I^2 = .%, p = .) 14.21 (8.23, 21.43) 15.23
MTX–naive cohorts
Lee (MTX–naive) (2022) 7.41 (2.92, 17.55) 4.25
Mahajan (2022) 0.00 (0.00, 5.92) 4.42
Kumar (2020) 7.84 (4.03, 14.72) 5.04
Talme (biologics) (2017) 3.12 (0.55, 15.74) 3.46
Gisondi (2016) 7.27 (2.86, 17.26) 4.27
Subtotal (I^2 = 59.34%, p = 0.04) 4.44 (1.17, 9.22) 21.44
Data not provided
BenLagha (2019) 5.68 (2.45, 12.62) 4.88
vanderVoort (2016) 8.11 (3.77, 16.58) 4.67
Subtotal (I^2 = .%, p = .) 6.74 (3.24, 11.26) 9.55
Heterogeneity between groups: p = 0.069
Overall (I^2 = 76.34%, p = 0.00); 9.66 (6.92, 12.75) 100.00
–50
0
50
100
(C)

Supplementary Figure 2: Forrest plots for pooled prevalence psoriasis of low risk of advanced liver fibrosis subgrouped based on (A) continent, (B) non-invasive tests, and (C) methotrexate user percentage

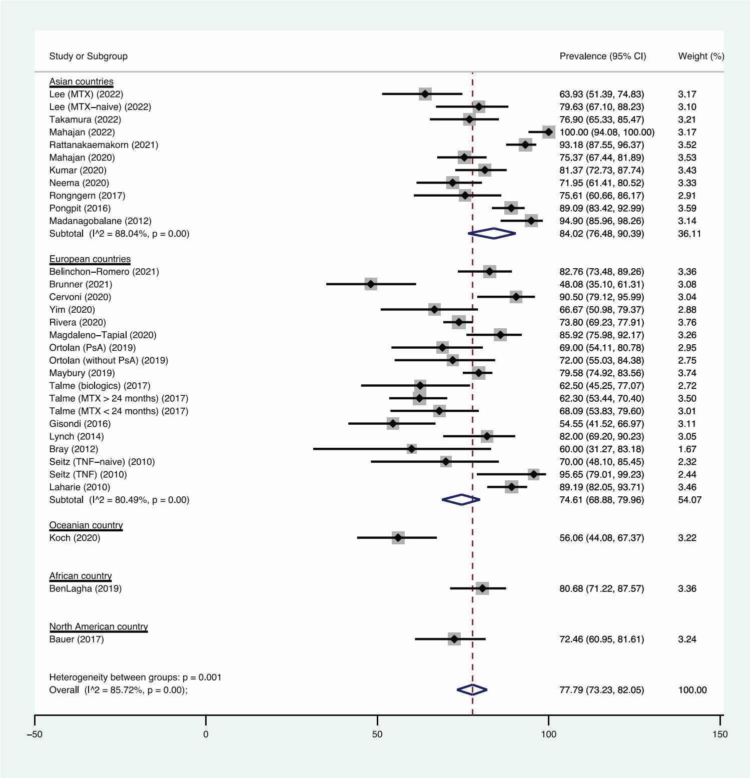

(A)

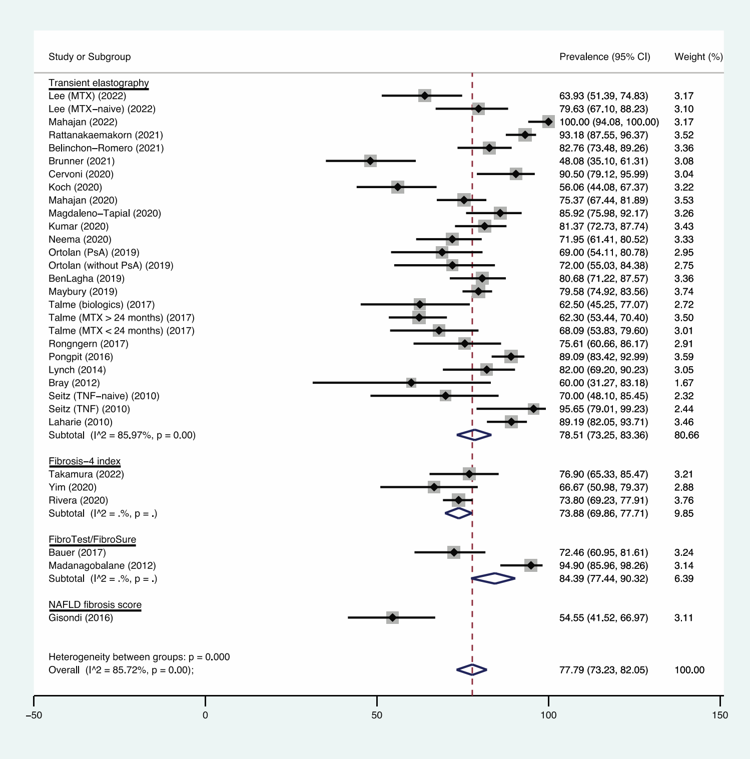

(B)

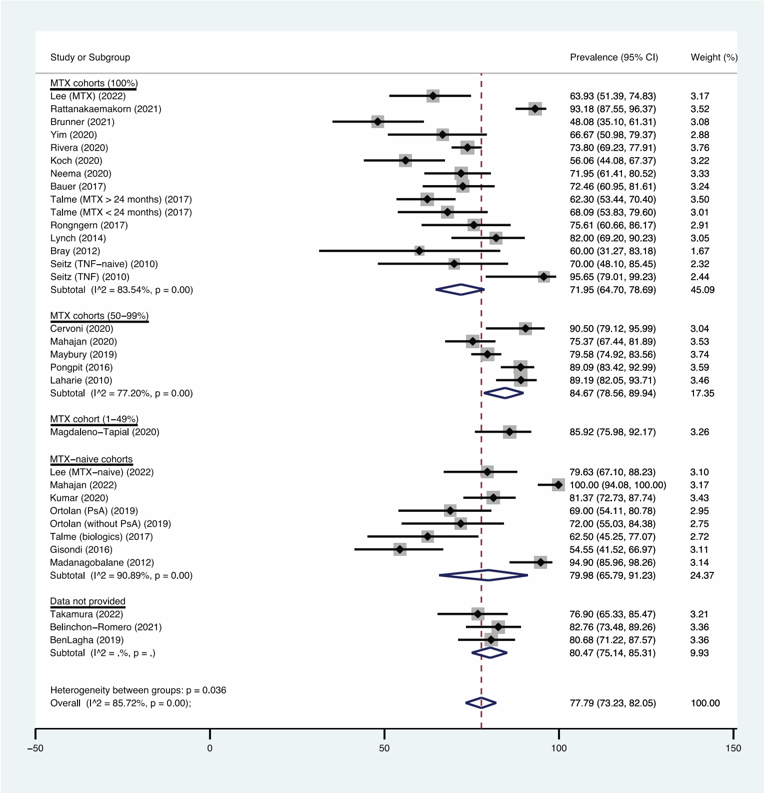
Study or Subgroup
Prevalence (95% CI)
Weight (%)
MTX cohorts (100%)
Lee (MTX) (2022) 63.93 (51.39, 74.83) 3.17
Rattanakaemakorn (2021) 93.18 (87.55, 96.37) 3.52
Brunner (2021) 48.08 (35.10, 61.31) 3.08
Yim (2020) 66.67 (50.98, 79.37) 2.88
Rivera (2020) 73.80 (69.23, 77.91) 3.76
Koch (2020) 56.06 (44.08, 67.37) 3.22
Neema (2020) 71.95 (61.41, 80.52) 3.33
Bauer (2017) 72.46 (60.95, 81.61) 3.24
Talme (MTX > 24 months) (2017) 62.30 (53.44, 70.40) 3.50
Talme (MTX < 24 months) (2017) 68.09 (53.83, 79.60) 3.01
Rongngern (2017) 75.61 (60.66, 86.17) 2.91
Lynch (2014) 82.00 (69.20, 90.23) 3.05
Bray (2012) 60.00 (31.27, 83.18) 1.67
Seitz (TNF−naive) (2010) 70.00 (48.10, 85.45) 2.32
Seitz (TNF) (2010) 95.65 (79.01, 99.23) 2.44
Subtotal (I^2 = 83.54%, p = 0.00) 71.95 (64.70, 78.69) 45.09
MTX cohorts (50−99%)
Cervoni (2020) 90.50 (79.12, 95.99) 3.04
Mahajan (2020) 75.37 (67.44, 81.89) 3.53
Maybury (2019) 79.58 (74.92, 83.56) 3.74
Pongpit (2016) 89.09 (83.42, 92.99) 3.59
Laharie (2010) 89.19 (82.05, 93.71) 3.46
Subtotal (I^2 = 77.20%, p = 0.00) 84.67 (78.56, 89.94) 17.35
MTX cohort (1−49%)
Magdaleno−Tapial (2020) 85.92 (75.98, 92.17) 3.26
MTX−naive cohorts
Lee (MTX−naive) (2022) 79.63 (67.10, 88.23) 3.10
Mahajan (2022) 100.00 (94.08, 100.00) 3.17
Kumar (2020) 81.37 (72.73, 87.74) 3.43
Ortolan (PsA) (2019) 69.00 (54.11, 80.78) 2.95
Ortolan (without PsA) (2019) 72.00 (55.03, 84.38) 2.75
Talme (biologics) (2017) 62.50 (45.25, 77.07) 2.72
Gisondi (2016) 54.55 (41.52, 66.97) 3.11
Madanagobalane (2012) 94.90 (85.96, 98.26) 3.14
Subtotal (I^2 = 90.89%, p = 0.00) 79.98 (65.79, 91.23) 24.37
Data not provided
Takamura (2022) 76.90 (65.33, 85.47) 3.21
Belinchon−Romero (2021) 82.76 (73.48, 89.26) 3.36
BenLagha (2019) 80.68 (71.22, 87.57) 3.36
Subtotal (I^2 = .%, p = .) 80.47 (75.14, 85.31) 9.93
Heterogeneity between groups: p = 0.036
Overall (I^2 = 85.72%, p = 0.00); 77.79 (73.23, 82.05) 100.00
−50
0
50
100
150

(C)

Supplementary Figure 3: Forrest plots of pooled odds ratios for the association of variables with liver fibrosis; (A) age (B) male (C) PASI > 10 (D) Psoriatic arthritis (E) Cumulative MTX dose > 1500 mg (F) BMI>30 (G) DM (H) HT (I) DLP (J) Metabolic syndrome

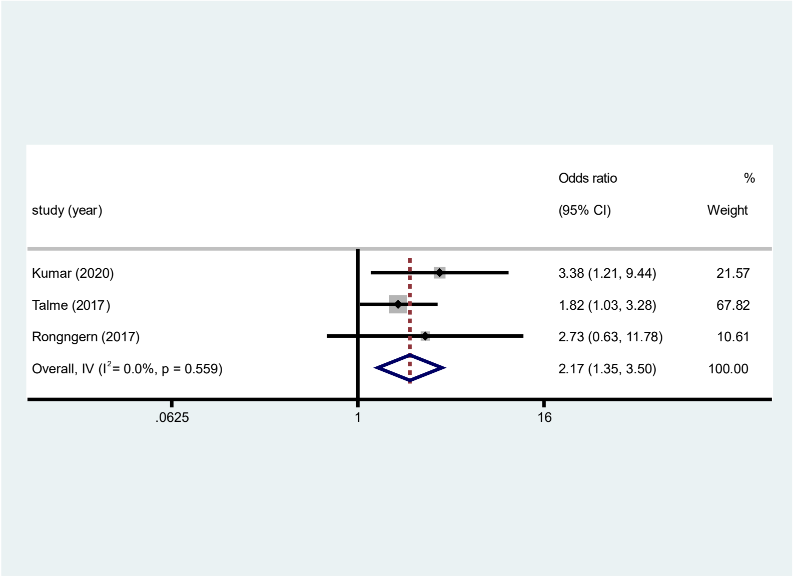

(A)

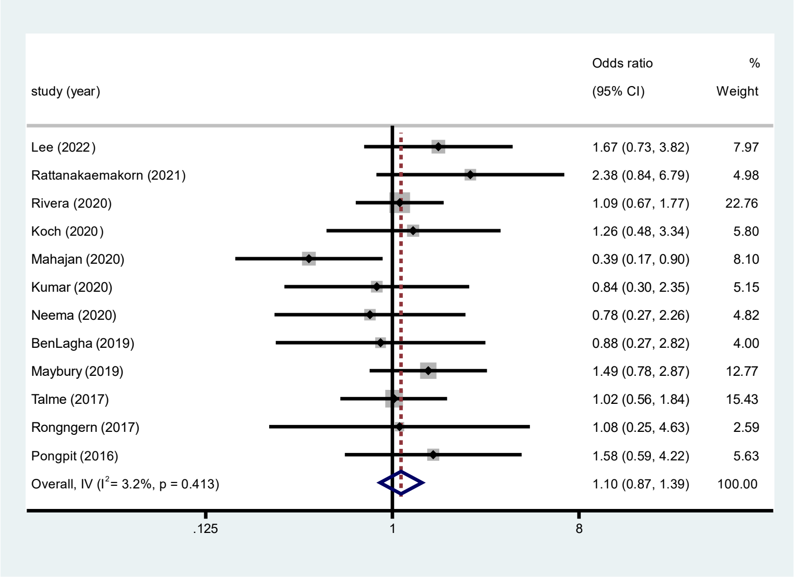

(B)

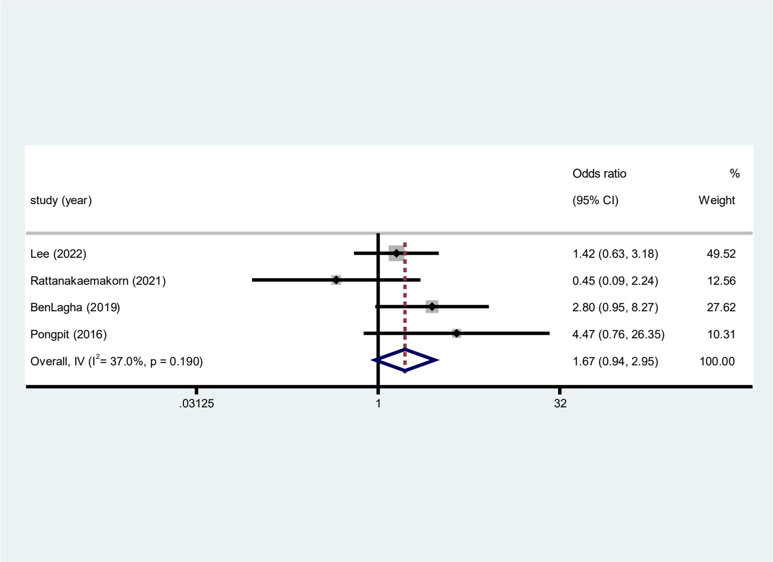

Odds ratio
%
study (year)
(95% CI)
Weight
Lee (2022)
1.42 (0.63, 3.18)
49.52
Rattanakaemakorn (2021)
0.45 (0.09, 2.24)
12.56
BenLagha (2019)
2.80 (0.95, 8.27)
27.62
Pongpit (2016)
4.47 (0.76, 26.35)
10.31
Overall, IV (I²= 37.0%, p = 0.190)
1.67 (0.94, 2.95)
100.00
.03125
1
32

(C)

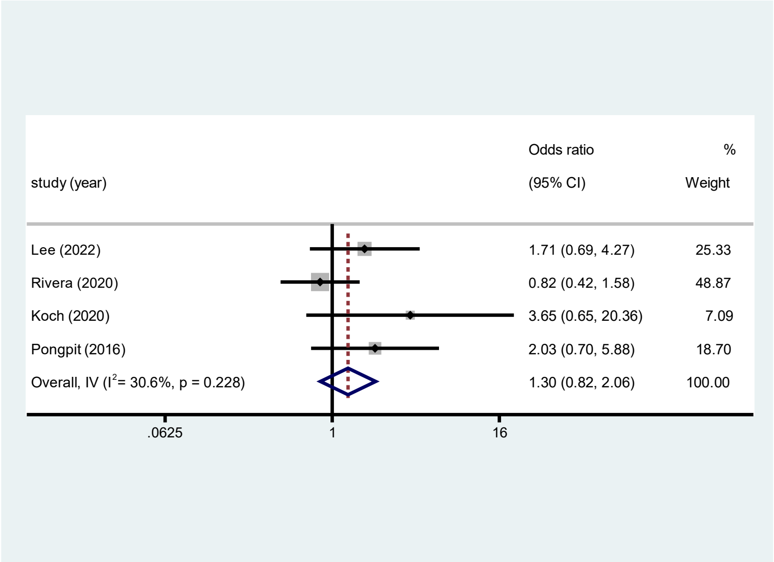

Odds ratio
%
study (year)
(95% CI)
Weight
Lee (2022)
1.71 (0.69, 4.27)
25.33
Rivera (2020)
0.82 (0.42, 1.58)
48.87
Koch (2020)
3.65 (0.65, 20.36)
7.09
Pongpit (2016)
2.03 (0.70, 5.88)
18.70
Overall, IV (I²= 30.6%, p = 0.228)
1.30 (0.82, 2.06)
100.00
.0625
1
16

(D)

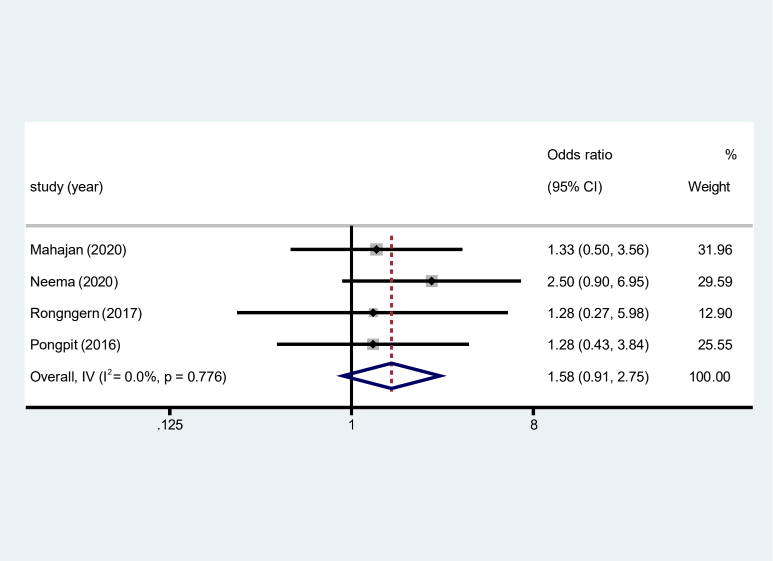

Odds ratio
%
study (year)
(95% CI)
Weight
Mahajan (2020)
1.33 (0.50, 3.56)
31.96
Neema (2020)
2.50 (0.90, 6.95)
29.59
Rongngern (2017)
1.28 (0.27, 5.98)
12.90
Pongpit (2016)
1.28 (0.43, 3.84)
25.55
Overall, IV (I²= 0.0%, p = 0.776)
1.58 (0.91, 2.75)
100.00
.125
1
8

(E)

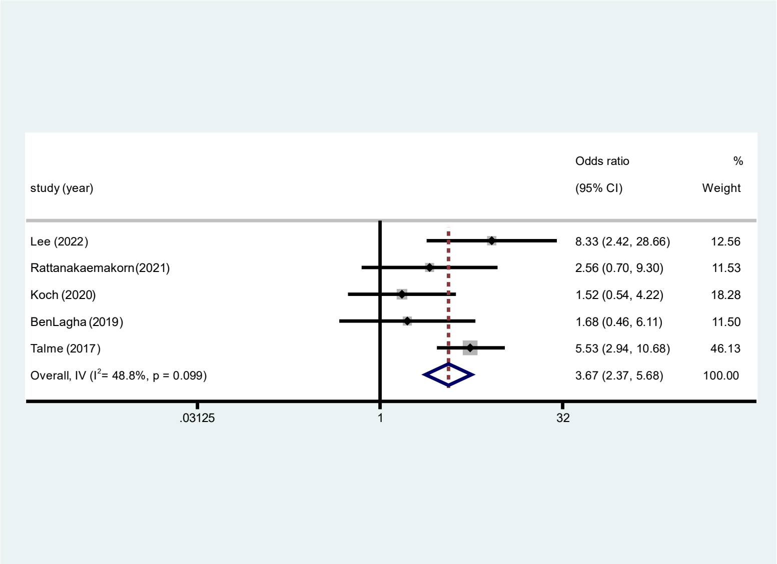
study (year)
Odds ratio (95% CI)
% Weight
Lee (2022) 8.33 (2.42, 28.66) 12.56
Rattanakaemakorn(2021) 2.56 (0.70, 9.30) 11.53
Koch (2020) 1.52 (0.54, 4.22) 18.28
BenLagha (2019) 1.68 (0.46, 6.11) 11.50
Talme (2017) 5.53 (2.94, 10.68) 46.13
Overall, IV (I²= 48.8%, p = 0.099) 3.67 (2.37, 5.68) 100.00
.03125
1
32

(F)

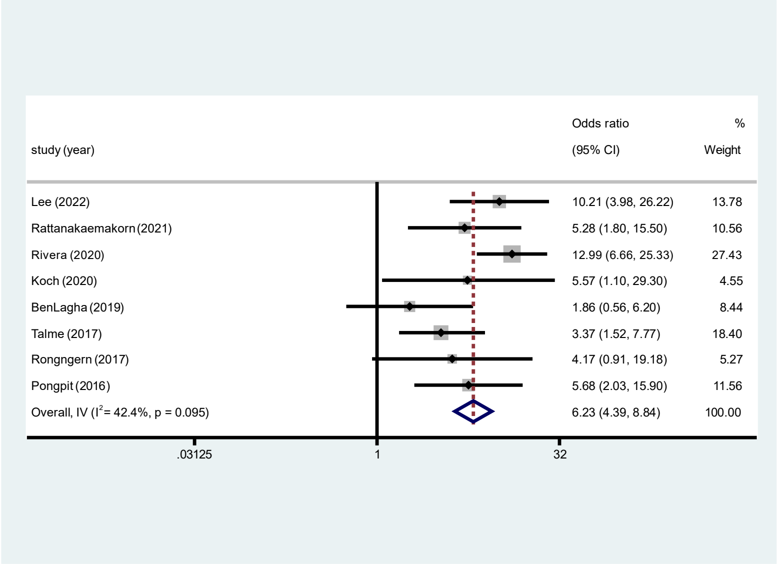
study (year)
Odds ratio (95% CI)
% Weight
Lee (2022) 10.21 (3.98, 26.22) 13.78
Rattanakaemakorn (2021) 5.28 (1.80, 15.50) 10.56
Rivera (2020) 12.99 (6.66, 25.33) 27.43
Koch (2020) 5.57 (1.10, 29.30) 4.55
BenLagha (2019) 1.86 (0.56, 6.20) 8.44
Talme (2017) 3.37 (1.52, 7.77) 18.40
Rongngern (2017) 4.17 (0.91, 19.18) 5.27
Pongpit (2016) 5.68 (2.03, 15.90) 11.56
Overall, IV (I²= 42.4%, p = 0.095) 6.23 (4.39, 8.84) 100.00
.03125
1
32

(G)

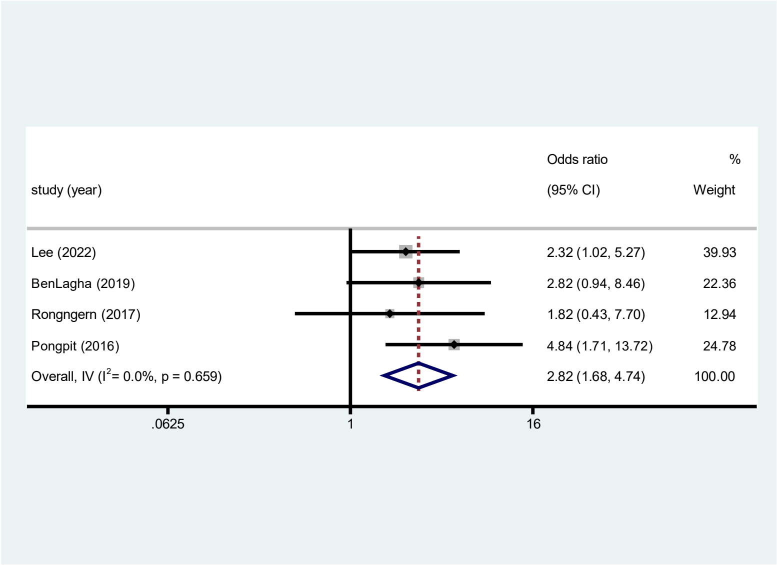
study (year)
Odds ratio (95% CI)
% Weight
Lee (2022) 2.32 (1.02, 5.27) 39.93
BenLagha (2019) 2.82 (0.94, 8.46) 22.36
Rongngern (2017) 1.82 (0.43, 7.70) 12.94
Pongpit (2016) 4.84 (1.71, 13.72) 24.78
Overall, IV (I²= 0.0%, p = 0.659) 2.82 (1.68, 4.74) 100.00
.0625
1
16

(H)

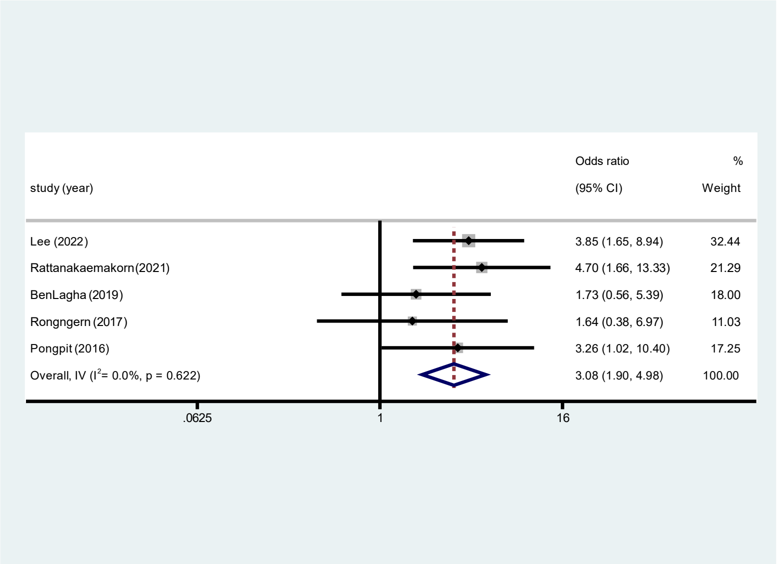
study (year)
Odds ratio (95% CI)
% Weight
Lee (2022) 3.85 (1.65, 8.94) 32.44
Rattanakaemakorn(2021) 4.70 (1.66, 13.33) 21.29
BenLagha (2019) 1.73 (0.56, 5.39) 18.00
Rongngern (2017) 1.64 (0.38, 6.97) 11.03
Pongpit (2016) 3.26 (1.02, 10.40) 17.25
Overall, IV (I^2= 0.0%, p = 0.622) 3.08 (1.90, 4.98) 100.00
.0625
1
16

(I)

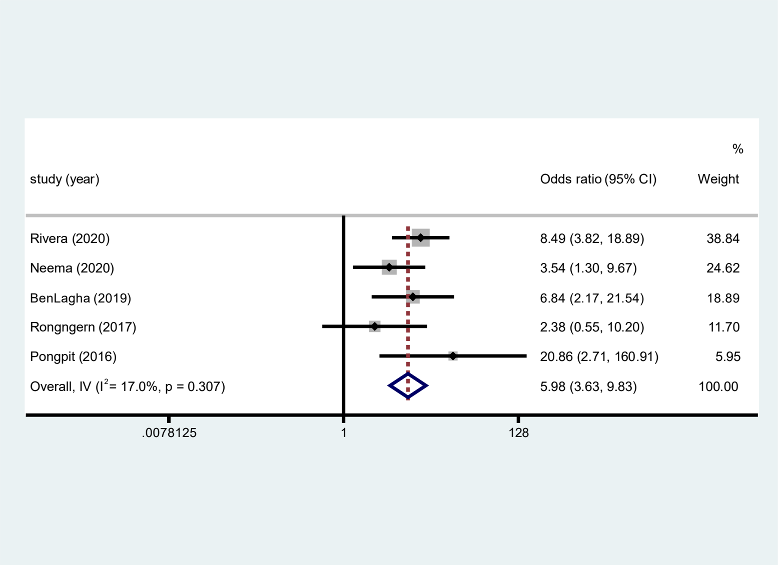
study (year)
Odds ratio (95% CI)
% Weight
Rivera (2020) 8.49 (3.82, 18.89) 38.84
Neema (2020) 3.54 (1.30, 9.67) 24.62
BenLagha (2019) 6.84 (2.17, 21.54) 18.89
Rongngern (2017) 2.38 (0.55, 10.20) 11.70
Pongpit (2016) 20.86 (2.71, 160.91) 5.95
Overall, IV (I^2= 17.0%, p = 0.307) 5.98 (3.63, 9.83) 100.00
.0078125
1
128

(J)
