# Supplementary material for: Liver fibrosis prevalence and risk factors in patients with psoriasis: A systematic review and meta-analysis
Source: Front Med (Lausanne). 2022 Dec 15;9:1068157. doi: 10.3389/fmed.2022.1068157 (PMC9797863; doi:10.3389/fmed.2022.1068157)
Supplement: Supplementary file 2 [file Table_1.pdf]

Supplementary Table 1: Search strategy

**PubMed**	("Psoriasis"[Mesh] OR "Arthritis, Psoriatic"[Mesh] OR psoria*) AND ("Liver Diseases"[Mesh] OR "Liver Cirrhosis"[Mesh] OR "End Stage Liver Disease"[Mesh] OR "Fatty Liver"[Mesh] OR "hepatic fibrosis")
**Embase**	1. 'psoriasis'/exp OR psoriasis<br>2. 'liver'/exp OR liver<br>3. 'fibrosis'/exp OR fibrosis<br>4. 'cirrhosis'/exp OR cirrhosis<br>5. 2 AND 3<br>6. 4 OR 5<br>7. 1 AND 6
**Scopus**	(TITLE-ABS-KEY (psoria*) AND TITLE-ABS-KEY ((liver AND fibrosis) OR (hepatic AND fibrosis) OR (cirrho*) OR (fatty AND liver)))
**Cochrane Library**	(Psoriasis OR psoria*):ti,ab,kw AND ("Liver fibrosis" OR "hepatic fibrosis" OR cirrho* OR "fatty liver" OR "NAFLD"):ti,ab,kw
