# Supplementary material for: Liver fibrosis prevalence and risk factors in patients with psoriasis: A systematic review and meta-analysis
Source: Front Med (Lausanne). 2022 Dec 15;9:1068157. doi: 10.3389/fmed.2022.1068157 (PMC9797863; doi:10.3389/fmed.2022.1068157)
Supplement: Supplementary file 3 [file Table_2.pdf]

Supplementary Table 2: Characteristics of included studies

Lee, 2022 (27)		
Methods	- Case-control study<br>- Malaysia	
Participants	MTX group (N=61)<br>▪ Age 52.98±15.5<br>▪ Female 29 (47.5)<br>▪ Alcohol 5 (8.2)<br>▪ Diabetes 13 (21.3)<br>▪ Hypertension 27 (44.3)<br>▪ Dyslipidemia 25 (41.0)<br>▪ BMI 27.0±6.2<br>▪ Obese 19 (31.1)<br>▪ Duration of psoriasis 11.9±6.7 years<br>▪ PASI score 15.8 (IQR 18.4)<br>▪ Psoriatic arthritis 19 (31.1)<br>▪ MTX cumulative dose ≥1 g; 1-1.5 gm = 5 (8.2%), 1.5-3 gm = 27(44.3%), 3-4.5 gm = 17 (27.8%), >4.5 gm = 12 (19.7%).<br>▪ NAFLD 6 (9.8)	MTX-naive group (N=56)<br>▪ Age 52.2±15.1<br>▪ Female 24 (42.9)<br>▪ Alcohol 5 (8.9)<br>▪ Diabetes 18 (32.1)<br>▪ Hypertension 23 (41.1)<br>▪ Dyslipidemia 19 (33.9)<br>▪ BMI 26.7±5.0<br>▪ Obese14 (25.0)<br>▪ Duration of psoriasis 10.4±9.4 years<br>▪ PASI score 4.3 (IQR 6.6)<br>▪ Psoriatic arthritis 8 (14.3)<br>▪ NAFLD 0 (0)
Investigations	▪ TE, FIB-4, APRI	
Outcomes	MTX group<br>- TE <6.5 kPa = 39 (63.9)<br>- FIB-4 <1.45 = 48 (78.7)<br>- APRI <0.7 = 54 (88.5)<br>- TE >11.5 kPa = 7 (11.5)<br>- FIB-4 >3.25 = 2 (3.3)<br>- APRI >1.0 = 5 (8.2)	MTX-naive group<br>- TE <6.5 kPa = 45 (80.4)<br>- FIB-4 <1.45 = 46 (82.1)<br>- APRI <0.7 = 49 (87.5)<br>- TE > 11.5 kPa = 4 (7.1)<br>- FIB-4 > 3.25 = 2 (3.6)<br>- APRI >1.0 = 6 (10.7)
	- Obesity adjusted OR 8.26 (1.73-39.43)<br>- Diabetes adjusted OR 30.35 (7.52-122.42)<br>- MTX use with deranged ALT adjusted OR 68.56 (8.26-568.86)	
Comments		

Mahajan, 2022 (30)	
Methods	- Cross-sectional, case–control study<br>- India
Participants	- 61 psoriasis patients<br>- Female 18 (29.5)<br>- Age 47.5±13.8<br>- Duration of psoriasis 3±12.5 years<br>- PASI <6/6-12/>12 = 48(78.7)/9(14.7)/4(6.5)<br>- Psoriatic arthritis 4 (6.5)<br>- Pregnant and lactating women and patients with erythroderma, having history of addictions, systemic medications that can induce fatty liver (methotrexate, retinoids, antiretroviral drugs, amiodarone, tamoxifen, corticosteroid, valproic acid), preexisting diabetes mellitus, hypertension, dyslipidemia, hepatitis B or hepatitis C virus infection, hepatic or renal disease, morbid obesity (body mass index [BMI] >30 kg/m2), or malignancy were excluded.
Investigations	- TE
Outcomes	- TE <7.6 kPa = 61 (100)
Comments	

Takamura, 2022 (28)	
Methods	- Retrospective study<br>- Japan
Participants	- 65 patients with psoriasis treated with IL-17i<br>- Age 46 (range 40-54.5)<br>- Female 16 (24.6)<br>- BMI 26.3 (range 23.7-29.9)<br>- Psoriasis subtypes Psoriasis vulgaris/psoriatic erythroderma/pustular psoriasis = 42(64.6)/4(6.2)/3(4.6)<br>- Psoriatic arthritis 16(24.6)<br>- Duration of psoriasis 12 (range 5–20) years<br>- PASI 8.7 (4.6–16.4)<br>- PASI <5/5-9/^{3}10 = 17(26.1)/18(27.7)/30(46.2)<br>- Alcohol intake 0/1-19/20-59/>60/no data = 26(40.0)/3(4.6)/2(3.1)/1(1.5)/33(50.8)<br>- Obesity 43 (66.2)<br>- Diabetes 15 (23.1)<br>- Hypertension 20 (30.8)<br>- Dyslipidemia 9 (13.8)
Investigations	- NFS, FIB-4
Outcomes	- NFS<-1.455 = 75.4%<br>- FIB-4 <1.3 = 76.9%
Comments	

Rattanakaemakorn, 2021 (31)	
Methods	- Retrospective cohort study<br>- Thailand
Investigations	- TE
Participants	- 132* psoriasis patients who are on MTX with/without acetretin<br>- Age 52±14.1<br>- Female 68(51.5)<br>- BMI 31.51±6.0<br>- PASI 8.38±6.42<br>- Diabetes 23(17.4)<br>- Obesity 53(40.2)<br>- Dyslipidemia 55(41.7)
Outcomes	- TE < 8 kPa = 123(93.2)<br>- TE ≥ 10 kPa = 7(5.3)<br>- DM adjusted HR 2.40 (1.05–5.51)<br>- Obesity adjusted HR 3.28 (1.18–9.16)<br>- Dyslipidemia adjusted HR 1.73 (0.67-4.47)
Comments	* Patient data was available for re-calculation (patients with viral hepatitis or have history of alcohol use were excluded)

Belinchón-Romero, 2021 (32)	
Methods	▪ Prospective cohort study<br>▪ Spain
Participants	91 moderate-to-severe psoriasis patients with NAFLD (87 of 91 patients had valid TE results)<br>- Female 32 (35.2)<br>- Age 53 (IQR 45.5–61.5)<br>- Duration of psoriasis 20 years (IQR 10–28)<br>- BMI ^{3}30 or obesity 54 (59.3)<br>- Abnormal waist circumference 8 (8.8)<br>- Smokers 23 (25.3)<br>- Diabetes 23 (25.3)<br>- Cardiopathy 10 (11.0)<br>- Dyslipidemia 57 (62.6)<br>- Metabolic syndrome 54 (59.3)<br>- Psoriatic arthritis 19 (20.9)<br>- PASI 3.4 (IQR 1–8)<br>- BSA 4 (IQR 1-8)<br>- Patients with treatment 29(31.8)<br>- Exclusion criteria; treatment with systemic corticosteroids in the previous 30 days, moderate alcohol consumption (> 3 units of alcohol per day in man or more than 2 units in women), other chronic liver disease (including B or C hepatitis-infected patients, Wilson's disease, autoimmune hepatitis, primary biliary cirrhosis, primary sclerosant cholangitis or hepatic malignancy), incapacitating disease or cognitive limitation, HIV-infected patients, and pregnancy
Investigations	▪ TE<br>▪ Liver biopsy, FIB-4, NFS (for 13 patients)
Outcomes	▪ (N=87) TE <7.8 kPa = 72(82.8)<br>▪ DM adjusted OR 5.24 (1.15-23.7)<br>▪ "ALT > 33 U/L" adjusted OR 16.49 (3.15-86.35)<br>▪ "ERS ≥ 16 mm" adjusted OR 11.93 (2.30-61.95)
Comments	

Brunner, 2021 (33)	
Methods	- Prospective cohort study<br>- Hungary
Participants	52 psoriasis patients undergoing methotrexate therapy<br>- Age 54.0±13.4 years<br>- Female 26(50)<br>- BW 91.6 ± 17.7 kg<br>- BMI 31.4 ± 5.1 kg/m2<br>- Body fat 36.3 ± 8.8 %<br>- Never smoked (n = 17, 32.7%), quit smoking (n = 21, 40.4%), smokes < 5 cigarettesper day (n = 4, 7.7%), smokes 5–10 cigarettes per day (n = 5, 9.6%), smokes 11–20 cigarettesper day (n = 4, 7.7%), smokes > 20 cigarettes per day (n = 1, 1.9%).<br>- MTX duration 4.8 ± 3.0 years<br>- MTX cumulative dose 2.2738 ± 2.2387 g
Investigations	- TE
Outcomes	▪ TE < 8.2 kPa = 72 (82.8)<br>▪ TE > 9.7 kPa = 14 (26.9)<br>▪ No correlation between the patients' liver stiffness values and the cumulative MTX doses. ($r^2 = 1.130\times 10^{-4}$, p = 0.803)<br>▪ Patients with higher BMI values, total body fat% and visceral fat scores were significantly more likely to present with higher hepatic stiffness values. BMI was a significant predictor of hepatic fibrosis in both genders. In males, body fat% (R = 0.578, p = 0.002) and, especially, visceral fat scores (R = 0.716, p < 0.001) had statistically significant correlation with stiffness scores, while in females only visceral fat scores were statistically significant predictors of the liver stiffness values (R = 0.452, p = 0.023), and body fat% was not (R = 0.187, p = 0.382).
Comments	

Cervoni, 2020 (34)	
Methods	▪ Prospective cohort study<br>▪ France
Participants	▪ 66 psoriasis patients 49 had valid TE results, 63 had Forns index results, 65 had APRI scores, 64 had FPI results, 64 had Hepascore results, 65 had FIB-4 indexes, 64 had NFS scores, 61 had FibroTest results, 64 had Fibrometer NAFLD results)<br>▪ Age 54±2<br>▪ Female 39%<br>▪ MTX treatment 71%<br>▪ MTX cumulative dose >1500 mg 30%<br>▪ Alcohol consumption 19±5<br>▪ Anti-HBc antibodies 7.6%<br>▪ HCV antibodies 1.5%<br>▪ ALT >UNL 27%<br>▪ Weight 78±2<br>▪ BMI 27.2 ± 0.7<br>▪ Waist circumference > 35 in (women) >40 in. (men) 50%<br>▪ Dyslipidemia 39%<br>▪ Hypertension 70%<br>▪ Diabetes 23%
Investigations	▪ TE, FibroTest/FibroSure, Forns index, APRI, FPI, Hepascore, FIB-4, NFS, FibroMeter NAFLD
Outcomes	▪ TE<7.1 kPa = 90.5%<br>▪ FibroTest<0.49 = 88.6%<br>▪ Hepascore<0.5 = 92.2%<br>▪ Forns index<6.9 = 90.5%<br>▪ FPI<0.8 = 87.5%<br>▪ FibroMeter<0.49 = 78.1%<br>▪ FIB-4>3.25 = 4.6%<br>▪ NFS>0.676 = 6.3%<br>▪ APRI>1.5 = 3.1%
Comments	

<table>
<tr><td colspan="2">Yim, 2020 (19)</td></tr>
<tr><td>Methods</td><td>▪ Retrospective medical record review
▪ Spain</td></tr>
<tr><td>Participants</td><td>▪ 39 patients with psoriasis with ≥3 months of MTX treatment
▪ Age 49.8
▪ Female 24(61.5)
▪ MTX cumulative dose 1.89±1.5 g
▪ MTX duration 238 wk
▪ Diabetes 7(17.9)
▪ Dyslipidemia 12(30.8)
▪ Biologics 26(66.7)
▪ Patients who were lost to follow-up, non-compliant, completed MTX treatment, or had missing laboratory values were excluded</td></tr>
<tr><td>Investigations</td><td>▪ FIB-4</td></tr>
<tr><td>Outcomes</td><td>▪ FIB-4<1.45 = 26(66.7)
▪ FIB-4>3.25 = 1(2.6)
▪ No association was found between FIB-4 score and cumulative MTX dose or treatment duration.
▪ However, the change in Fib-4 score over treatment interval was associated with cumulative MTX dose (P = 0.03). Diabetes (P < 0.03), hyperlipidemia (P < 0.05), and statin use (P < 0.02) were found to be associated with higher Fib-4 scores.
▪ 8 patients completed a fibroscan, which demonstrated that a higher Fib-4 score was associated with increased median liver stiffness (P = 0.0002).
▪ 2 patients with no hepatotoxic risk factors and persistently normal LFTs had high-risk Fib-4 scores of 4.39 and 4.6.</td></tr>
<tr><td>Comments</td><td></td></tr>
</table>

Rivera, 2020 (35)	
Methods	- Retrospective cross-sectional study<br>- Spain
Participants	- 457 patients (280 with NFS results, and 392 with FIB-4 results) who are treated with MTX<br>- Age 53.3 ± 14.0<br>- Female 199 (43.5)<br>- BMI 28 ± 5.1<br>- BMI ≥30 = 134 (29.3)<br>- Smoking > 10 daily cigarettes consumption = 88 (19.3)<br>- Alcohol > 20, 40g of daily alcohol consumption in female and male = 13 (2.8)<br>- Obesity 203 (44.4)<br>- Dyslipidemia 123 (26.9)<br>- Diabetes 115 (25.2)<br>- Hypertension 116 (25.4)<br>- Patients who had been previously or simultaneously treated with immunomodulatory biologics were excluded from the study, as well as those with severe concomitant diseases that could affect the evaluation of psoriasis impact in HRQoL (e.g., cancer, psychiatric diseases, other skin diseases), those participating in a clinical trial at the time of the study, or who presented other types of psoriasis
Investigations	- FIB-4, NFS
Outcomes	▪ FIB-4 <1.3 = 73.8%<br>▪ NFS <-1.455 = 62.8%
Comments	

Koch, 2020 (36)	
Methods	▪ Retrospective<br>▪ New Zealand
Participants	- 66 psoriasis patients on MTX<br>- Female 34 (51.5)<br>- Age 51.2 (SD 14.0)<br>- MTX cumulative dose 5.7 (SD 7.1) g<br>- MTX cumulative dose > 3.5 g 32 (48.5)<br>- MTX dose/wk 18.1 (SD 5.3) mg<br>- Duration of therapy 72.5 (SD 60.8) months<br>- BMI 32.1 (SD 7.1)<br>- Obesity (BMI>30) 42 (63.6)<br>- Morbid obesity (BMI>35) 20 (30.3)<br>- Diabetes 9 (13.6)<br>- HPT 18 (27.3)<br>- Psoriatic arthritis 7 (10.6)<br>- PIIINP 4.6 (SD 2.0)<br>- PASI 3.9 (SD 4.2)
Investigations	- TE, PIIINP<br>- Liver biopsy performed in 2 patients (3)
Outcomes	- TE <7.1 kPa = 37(56.1)<br>- TE >9 kPa = 23(34.8)<br>- PIIINP >4.2 µg/L = 29(43.9)
Comments	

Mahajan, 2020 (29)	
Methods	▪ Prospective, cross-sectional study<br>▪ India
Participants	- 134 psoriasis patients<br>- Age 44.13±13.86 years<br>- Age of onset 33.09 ± 14.768 years<br>- Female 40(30.3)<br>- Alcohol intake (any amount) 42(31.3)<br>- Smoking (any number) 32(23.9)<br>- Acitretin 42 patients, cyclosporine 10, hydroxyurea 5<br>- MTX exposure 103(76.9)<br>- MTX cumulative dose 0.89 ± 1.12 g (range 0-5.2 g)<br>- Hypertension 27(20.1)<br>- Diabetes 12(9.8)<br>- Weight 69.06 ± 13.55 kg<br>- Height 164.14 ± 9.23 cm<br>- BMI 25.64 ± 4.34 kg/m^2<br>- WC 95.01 ± 12.25 cm<br>- BSA 17.25 ± 19.9 %<br>- PASI 8.87 ± 6.7 %<br>- Metabolic syndrome 56(41.8)
Investigations	- TE
Outcomes	▪ TE <7 kPa = 101(75.4)<br>▪ TE ≥9 kPa = 16(6.7)<br>▪ Neither methotrexate exposure nor total cumulative dose of ≥1.5 was associated with significant fibrosis.<br>▪ Female sex (P = 0.024) and the presence of metabolic syndrome (P = 0.034) were the two variables associated with significant liver fibrosis. ORs for the female gender and metabolic syndrome was estimated to be 2.51 (95%CI:1.09–5.81) and 2.33 (95%CI:1.03–5.27), respectively.<br>▪ APRI, NFS and FIB-4 had low sensitivity in comparison to transient elastography.
Comments	

Magdaleno-Tapial, 2020 (37)	
Methods	▪ Prospective, cross-sectional study<br>▪ Spain
Participants	- 71 psoriasis patients<br>- Female 24(33.8)<br>- Age 46.7±14 years<br>- Duration of psoriasis 21±13 years<br>- Maximum historical PASI 14.4±6.5<br>- Maximum historical BSA 24%<br>- Baseline PASI 2±3.3<br>- Psoriatic arthritis 14(20.3)<br>- Treatment at the beginning of the study (n; %); Methotrexate 7(10.1), Ciclosporin 2(2.9), Apremilast 3(4.3), Biological drug 55(71.4)<br>- Comorbidities; smoking 24(36.4), diabetes 14(20.6), hypertension 13(19.1), dyslipidemia 23(33.8), metabolic syndrome 25(36.8)<br>- Weight 84.1±19.8<br>- Height 171±10.1<br>- WC 103.2±17.2<br>- BMI 28.6±6
Investigations	- TE
Outcomes	- TE < 7.7 kPa = 61(85.9)<br>- TE ≥ 9.5 kPa = 6(8.5)
Comments	

Kumar, 2020 (38)	
Methods	- Cross-sectional study<br>- India
Participants	- 102 adults with psoriasis who were not treated with methotrexate.<br>- Mean age 42.12<br>- Females 61(59.8)<br>- Exclusion: pregnant women, those with ascites, past liver disease, and patients having active medical device implants
Investigations	- TE
Outcomes	- TE < 7.5 kPa = 83(81.3)<br>- TE ≥ 10 kPa = 8(7.8)
Comments	

Neema, 2020 (39)	
Methods	- Cross-sectional study<br>- India
Participants	82 psoriasis patients<br>- Age 47.04±12.45<br>- Female 20(24.4)<br>- Psoriasis duration 10.01±8.68<br>- BMI 25.16±3.95<br>- Waist circumference 93.03±9.28<br>- DBP 81.16±7.19<br>- Sugar (F) 94.66±29.56<br>- Sugar (PP) 129.51±47.48<br>- AST 29.93±18.36<br>- PASI 16.26±7.99<br>- PGA 3.76±1.05<br>- MetS 32(39)<br>- MTX cumulative dose <1.5 g = 60 (73.2)<br>- MTX cumulative dose >1.5 g = 22 (26.8)
Investigations	- TE
Outcomes	- TE < 7 kPa = 59(72.0)<br>- Value of >7 kPa was significantly associated with age, waist circumference, diastolic blood pressure, fasting and post prandial bloodsugar, AST, PASI and presence of metabolic syndrome. Cumulative methotrexate dose was notsignificantly associated with high TE value. Mean TE value in patients with metabolic syndromewas significantly higher.
Comments	

<table>
<tr><td colspan="3">Ortolan, 2019 (40)</td></tr>
<tr><td>Methods</td><td colspan="2">- Prospective cohort study
- Italy</td></tr>
<tr><td>Investigations</td><td colspan="2">- TE</td></tr>
<tr><td rowspan="2">Participants</td><td colspan="2">- Exclusion criteria: liver diseases (other than NAFLD) potentially causing LF, alcohol consumption ≥ 20 g/day, daily use of non-steroidal anti-inflammatory drugs for a period ≥1 month in the past year, current use of methotrexate, or use of methotrexate in the previous year and other overlapping rheumatic diseases</td></tr>
<tr><td>- Psoriasis arthritis (N=43)
- Age 60.2 ± 8.4
- Female 11(25.6)
- PsA duration 12.6 ± 8.5
- Psoriasis duration 20 ± 12.2 years
- PASI 1.5 ± 2.5
- CRP 0.5 ± 0.7
- BMI 25.7 ± 3.4
- WC 99.7 ± 12.1
- DM 16%
- HT 46%
- MetS 35%
- MTX 0%</td><td>- Psoriasis without PsA (N=33)
- Age 54.5 ± 19.6
- Female 12 (36.4)
- Psoriasis duration 18.2 ± 14.2 years
- PASI 5±4.6
- CRP 0.7 ± 0.9
- BMI 29.1 ± 6.3
- WC 103.1 ± 13.1
- DM 21%
- HT 33%
- MetS 33%
- MTX 0%</td></tr>
<tr><td rowspan="2">Outcomes</td><td>- TE < 7 kPa = 69%</td><td>- TE < 7 kPa = 72%</td></tr>
<tr><td colspan="2">- NAFLD was more frequent in PsO (65% vs 35%, p = 0.044).
- In multivariable models with NAFLD and LF grading as outcomes, HOMA was independently associated with both (OR 1.34; 95%CI 1.06, 1.69; beta 0.88; 95%CI 0.54, 1.21, respectively).
- Female sex was independently associated with LF grading (OR 1.38 ; 95%CI 0.36, 5.29; 0.63 beta 1.81; 95%CI 0.05, 3.57).</td></tr>
<tr><td>Comments</td><td colspan="2"></td></tr>
</table>

BenLagha, 2019 (41)	
Methods	- Tunisia
Participants	- 88 patients with psoriasis and valid TE measurements<br>- Female 48(42.9)<br>- Age 45.6±14.3 years<br>- BMI 26.2±41 kg/m^2<br>- Overweight 60.9%, Obese 17%, Central obesity 67%<br>- Diabetes 20.5%<br>- Hypertension 28.4%<br>- Dyslipidemia 26.1%<br>- Metabolic syndrome 40.9%<br>- PASI score 10.4±9.1<br>- Severe psoriasis 65.9%<br>- MTX cumulative dose<br>- Psoriasis duration 12.64±11.52<br>- Exclusion: pregnant women, patients with known liver diseases, heavy alcohol drinkers and regular users of hepatotoxic medications other than methotrexate. Psoriasis was defined as severe in the fol-lowing conditions: Psoriasis Area and Severity Index (PASI)≥10, or generalized pustular, erythrodermic or arthropathic psoriasis, or requirement of a systemic medication.
Investigations	- TE
Outcomes	- TE <7 kPa = 71(80.7)<br>- TE >9.5 kPa = 5(5.7)
Comments	

Maybury, 2019 (42)	
Methods	- Prospective, cross-sectional study<br>- United Kingdom
Participants	- 400 psoriasis patients (333 had valid TE results)<br>- Severe psoriasis 100%<br>- Age 49.5±13 years<br>- Female 108(27.2)<br>- Weight 88±20 kg<br>- BMI 29.2±7 kg/m^2<br>- WC 102±16 cm<br>- Obesity 135(37.2)<br>- Diabetes 79(22)<br>- HOMA-IR 2.2(IQR 1.3-3.8)<br>- Alcohol 5(IQR 3-6) U/wk<br>- PASI 3.6(IQR 3.10-4.10)<br>- Current MTX use 85(21)<br>- History of MTX use 340(85)<br>- MTX dose 15 (IQR 13.6-15.5) mg/wk<br>- MTX duration 0.6 (IQR 0-2.4) years
Investigations	- TE
Outcomes	- TE < 7 kPa = 265(79.6)<br>- TE ≥ 8.7 kPa = 47(14.1)
Comments	

vandenReek, 2017 (55)		
Methods	- Netherlands	
Participants	Elevated PIIINP (N=41)<br>- Age 55.9±16.5<br>- Female 20(48.8)<br>- Alcohol use<br>o No 19(46.3)<br>o 0-20 13(31.7)<br>o >21 unit -<br>o Amount unknown 4(9.8)<br>- Malignancy 4(9.8)<br>- Diabetics 5(12.2)<br>- Duration of MTX use 3.7 (0.7-33.2) years<br>- Cumulative dose of MTX 2.747 (0.33-24.253) mg	No elevated PIIINP (N=142)<br>- Age 48.3±15.8<br>- Female 61(43.0)<br>- Alcohol use<br>o No 42(29.6)<br>o 0-20 53(37.3)<br>o >21 unit 3(2.1)<br>o Amount unknown 5(3.5)<br>- Malignancy 7(4.9)<br>- Diabetics 11(7.7)<br>- Duration of MTX use 3.3 (0.5-36.7) years<br>- Cumulative dose of MTX 2.270 (0.280-20.180) mg
Investigations	- PIIINP<br>- Liver biopsy	
Outcomes	- elevated PIIINP = 41 (22.4)<br>- 20 (49%) were referred to a hepatologist and/or underwent further diagnostics, and 6 of the 20 referred patients showed no liver abnormalities on biopsy, ultrasound or Fibroscan. Relevant liver problems consisted of Roenigk 3b (n = 1) and Roenigk 3a (n = 1) detected by liver biopsy, and high liver stiffness detected with Fibroscan (n = 2).<br>- 21 of 142 with normal PIIINP underwent further investigations; highest fibrosis score found was Roenigk 2–3a in 1 of 18 patients. Of note, 1 patient in this group had a Fibroscan stage F2, but liver biopsy showed Roenigk 1.	
Comments		

vanderVoort, 2017 (54)		
Methods	- Cross-sectional	
Investigations	- ELF, PIIINP	
Participants	- 119 PSO<br>- Age 49.8±14.3 years<br>- Female 45(37.8)<br>- BMI 27.2±5.8<br>- Disease duration; 20.1±14.5 years<br>- Severe psoriasis 15(12.5)<br>- PASI; 5.9±5.8<br>- MTX 18 (15.1)<br>- Diabetes 16 (12.1)<br>- Hypertension 40 (22.2)<br>- Dyslipidemia 80 (44.7)<br>- Alcohol intake (drinks/day) none/≤3/>3 = 43.2/47.7/9<br>- Smoking never/former/current = 27.7/29.4/42.9<br>- Median values (IQR) of ELF 8.96 (1.20)<br>- Median values (IQR) of PIIINP 7.56 (2.92)	- 151 PsA<br>- Age 52.8±11.7 years<br>- Female 70(46.3)<br>- BMI 26.5±4.2<br>- Disease duration; 9.9±9.3 years<br>- Severe psoriasis 2(1.3)<br>- PASI; 1.5±2.4<br>- MTX 69 (45.7)<br>- Diabetes 8 (7.3)<br>- Hypertension 38 (24.7)<br>- Dyslipidemia 38 (24.7)<br>- Alcohol intake (drinks/day) none/≤3/>3 = 30.9/66.2/2.9<br>- Smoking never/former/current = 40.5/42.5/17<br>- Median values (IQR) of ELF = 8.93 (0.98)<br>- Median values (IQR) of PIIINP = 7.23 (2.8)
Outcomes	- ELF > 9.8 = 25(21)<br>- PIIINP > 12.2 = 7(6)<br>- PIIINP > 15.3 = 6(5.2)	- ELF > 9.8 = 20(13.2)<br>- PIIINP > 12.2 = 9(6)<br>- PIIINP > 15.3 = 2(1.3)
Comments		

Bauer, 2017 (43)	
Methods	- A retrospective descriptive analysis<br>- United States
Participants	- 107 psoriasis patients undergoing treatment with MTX (69 had NASH FibroSure testing prior to starting MTX)<br>- Age 83.3±13.5 years<br>- Female 57(53.2)<br>- Non-hispanic white 107(100)<br>- BMI <28/28-30/>30 = 3(2.8), 17(15.9). 87(81.3)<br>- Diabetes 17(15.9)<br>- CKD stage III 4(3.7)<br>- Patients were excluded if they were younger than 18 years of age, had received MTX therapy for another indication, had received less than a 500-mg cumulative dose of MTX, or had been taking MTX for fewer than 90 days.
Investigations	- FibroSure (FibroTest)
Outcomes	- FibroSure not elevated = 50(71.5)<br>- 54 (78.3%) had elevated steatosis scores.<br>- Among the 107 patients who underwent NASH FibroSure testing during MTX therapy, the cumulative MTX dose corresponded to a statistically significant association of a higher NASH FibroSure hepatic fibrosis score in women (Spearman ρ = 0.21; *P* = .02) but not in men (Spearman ρ = 0.17; *P* = .11).<br>- All patients in the cohort except 1 were managed without a liver biopsy.
Comments	

<table>
<tr><td colspan="4">Talme, 2017 (44)</td></tr>
<tr><td>Methods</td><td colspan="3">- Prospective, cohort study
- Sweden</td></tr>
<tr><td>Investigations</td><td colspan="3">- TE</td></tr>
<tr><td rowspan="2">Participants</td><td colspan="3">- 201 psoriasis patients divided into 3 groups:</td></tr>
<tr><td>Biologics (N=32)
- Female 6(18.8)
- Age 48(18-76)
- Duration of psoriasis 20(21-38)
- BMI 27(21-38)
- BMI>25 25(75)
- BMI >30 13(41)
- DM 7(21.8)</td><td>MTX > 24 months (N=122)
- Female 52(41.9)
- Age 60(22-82)
- Duration of psoriasis 27(5-66)
- BMI 28(19-42)
- BMI>25 82(68)
- BMI>30 37(31)
- BMI>40 1(0.8)
- DM 20(16.1)
- MTX duration 60(25-432)</td><td>MTX ≤ 24 months (N=47)
- Female 17(34.7)
- Age 50(20-76)
- Duration of psoriasis 24(4-53)
- BMI 27(20-40)
- BMI>25 31(69)
- BMI>30 15(33)
- DM 5(10.2)
- MTX duration 16(1-24)</td></tr>
<tr><td rowspan="2">Outcomes</td><td>- TE <6.5 kPa = 20(62.5)
- TE >11.5 kPa = 1(3.1)</td><td>- TE <6.5 kPa = 76(62.3)
- TE >11.5 kPa = 11(9)</td><td>- TE <6.5 kPa = 32(68.1)
- TE >11.5 kPa = 3(6.4)</td></tr>
<tr><td colspan="3">- BMI >30 and diabetes were the strongest predictors for liver fibrosis
- BMI >30 associated with TE >6.5 kPa and >11.5 kPa with ORs of 5.53 (95CI: 2.94–10.68) and 11.42 (95CI: 4.00–41.24), respectively.
- Diabetes associated with TE >6.5 kPa and >11.5 kPa with ORs of 3.37 (95CI: 1.52–7.77) and 4.42 (95CI: 1.59–11.76), respectively,</td></tr>
<tr><td>Comments</td><td colspan="3"></td></tr>
</table>

Rongngern, 2017 (45)	
Methods	- Retrospective study<br>- Thailand
Participants	- 41 psoriasis patients who had received methotrexate<br>- Female 17(41.5)<br>- Age 51.2±11.6 years<br>- Psoriasis duration 12.7±5.9 years<br>- MetS 43.9%<br>- DLP 42.5%<br>- HT 39.3%<br>- DM 11(26.8)<br>- BMI 24.9±4.6 kg/m^2<br>- MTX cumulative dose 1.65 (range 0.8-8)<br>- MTX duration 6.4±3.2 years<br>- 2 (4.9%) occasionally drank alcohol during methotrexate treatment
Investigations	- TE<br>- Liver biopsy
Outcomes	- TE < 7.1 kPa = 31(75.6)<br>- TE ≥ 10 kPa = 3(7.3)<br>- TE ≥ 7.1 kPa; [Roenigk≥3a] sensitivity 50%, specificity 83.9%, PPV 50%, NPV 83.9%, accuracy 75.6%; [Metavir≥F2] sensitivity 50%, specificity 76.9%, PPV 10%, NPV 96.8%, accuracy 75.6%<br>- Ten of 41 patients developed methotrexate-induced liver injury (Roenigk grade≥3a) and two of them had significant liver fibrosis (Metavir fibrosis stage ≥2).
Comments	

Pongpit, 2016 (46)	
Methods	- Prospective, cross-sectional study<br>- Thailand
Participants	- 165 patients with psoriasis<br>- Age 49.2±14<br>- Female 90(54.5)<br>- BMI 24.8±4.7 kg/m^2<br>- Overweight 32.1%<br>- Obesity 20(12)<br>- WC 87.0±12.3 cm<br>- DLP 88(53.3)<br>- HT 55(33.3)<br>- DM 31(18.8)<br>- MetS 83(50.3)<br>- Duration of disease (years)†16.5±12.1<br>- PASI 3.0±2.7<br>- PASI >10 = 6(3.2)<br>- MTX cumulative dose; 0gm 57(34.5), 0-1.5gm 69(41.8), >1.5gm 39(23.5)<br>- TE 5.3 ± 2.9 kPa
Investigations	- TE
Outcomes	- TE <7 kPa = 147(89.1)<br>- TE > 9.5 kPa = 11(6.7)<br>- Two patients with LSM of 8.9 and 11 kPa underwent liver biopsy. The pathological reports revealed that Metavir fibrotic staging of F2 and F3 without hepatitis was confirmed.<br>- Waist circumference (OR: 1.24; 95% CI: 1.11–1.38; *P* = 0.0002), diabetes (OR: 12.70; 95% CI: 1.84–87.70; *P* = 0.010), and AST (OR: 1.08; 95% CI: 1.02–1.16; *P* = 0.017) were associated with high LSM.
Comments	

Gisondi, 2016 (47)	
Methods	- Cross-sectional study<br>- Italy
Participants	- 124 patients with chronic plaque psoriasis (55 with NFS results)<br>- Female 55 (44)<br>- Age 55±12 years<br>- Disease duration 21±13 (1–63) years<br>- Age of onset 34±15 (1–73) years<br>- Hypertension 41 (33%)<br>- Diabetes 23 (18%)<br>- PASI 13±10 (1–58)<br>- BMI 27.2±5<br>- Psoriatic arthritis 37 (29.8%)<br>- Dyslipidemia 13 (11%)<br>- AST 29±22<br>- ALT 35±34<br>- PLT 237±60<br>- Albumin 42±4<br>- NFS -1.57±1.4<br>- Liver steatosis 73 (59%)<br>- NAFLD 55 (44%)<br>- Alcohol; abstainers/ 1-2 drinks per day/ > 3 drinks per day = 33 (38%)/ 42 (48%)/ 12 (14%)<br>- Exclusion: Patients who had any clinical evidence of malignancy, cirrhosis or other secondary causes of chronic liver disease (i.e., autoimmune hepatitis, regular use of potentially hepatotoxic medications such as methotrexate, isoniazid, alpha-methyldopa, amiodarone or chemotherapies and a history of excessive alcohol consumption) were excluded
Investigations	- NFS
Outcomes	- NFS < -1.455 = 30 (54.5)<br>- NFS > 0.676 = 4 (7.3)<br>- Psoriasis resulted in a significant predictor of advanced liver fibrosis independently of age, sex, BMI, hypertension and diabetes in the multivariate analysis
Comments	

vanderVoort, 2016 (53)	
Methods	- Prospective, case-control study<br>- Netherlands
Participants	- 74 psoriasis patients with reliable TE scan<br>- Age 71.2±6.5 years<br>- Female 44.6%<br>- BMI 26.6 (24.1-28.5) kg/m^2<br>- Alcohol intake 7.5(IQR 0.9-7.5) U/wk<br>- Viral hepatitis 1.4%<br>- Hepatotoxic medication 2.7%<br>- Smoking never/former/current = 27.4/60.3/12.3<br>- MetS 52.1%<br>- FBS>100 or on DM medication 41.4%<br>- Waist circumference >88cm(female) >102cm(male) 45.8%<br>- TG >150 or on DLP medication 41.1%<br>- HDL < 40mg/dl(female) < 50 mg/dl(male) or on DLP medication 37.5%<br>- BP≥130/85 or on HT medication 88.1%<br>- Psoriasis duration 11.2 (IQR 15.8) years<br>- PASI 2 (IQR 3.2)
Investigations	- TE
Outcomes	- TE > 9.5 kPa = 6(8.1)
Comments	

Martyn-Simmons, 2014 (65)	
Methods	- Retrospective cohort study<br>- United Kingdom
Participants	- 27 chronic plaque psoriasis patients<br>- Age 56 ± 2.7 (26-77)<br>- Female 9 (33%)<br>- Comorbidities; diabetes 7 (26%), hypercholesterolemia 7 (26%), ischemic heart disease 5 (19%)<br>- Current smoker 7(26%), ex-smoker 5(19%), non-smoker 10(37%)<br>- Alcohol intake > 20 units/week 2 (8%)<br>- Mean weight 91 kg (range 49-130, SEM 4.3)<br>- Mean weekly dose of MTX 14.44 mg (range 5-30, SEM 1.17)<br>- Mean cumulative dose of MTX 7.53 g (range 0.87-22.36, SEM 1.30)
Investigations	- PIIINP > 4.2 μg/L<br>- ELF<br>- Liver biopsy
Outcomes	- AUROCs for mild fibrosis of serial and serial ELF were 0.589 (95CI: 0.379–0.800) and 0.643 (95CI: 0.391–0.895), respectively.<br>- AUROCs for at least moderate fibrosis of serial and serial ELF were 0.576 (95CI: 0.237–0.916) and 0.674 (95CI: 0.421–0.927), respectively.<br>- AUROCs for mild fibrosis of single PIIINP and single ELF were 0.582 (95CI: 0.363–0.801) and 0.693 (95% CI 0.482–0.904), respectively.<br>- AUROCs for at least moderate fibrosis of single PIIINP and single ELF were 0.667 (95% CI 0.363– 0.971) and 0.806 (95% CI 0.564–1.000)
Comments	

Lynch, 2014 (48)	
Methods	- Retrospective study<br>- Ireland
Participants	- 77 psoriasis on MTX (50 patients (65%) had a valid TE assessment, 70 had analyzed FibroTest results, 51 had serial PIIINP 1 year before TE)<br>- Female 36(46.8)<br>- Age 51(range 22-85)<br>- Psoriasis type; CPP 70(91), palmoplantar pustulosis 7(9)<br>- Psoriatic arthritis 20(26)<br>- BMI 27.7(range 19.8-63.6)<br>- Overweight 50(65)<br>- Obesity 24(31)<br>- Morbid obesity (BMI>40) 2(3)<br>- Alcohol consumption 0.06(0-24) U/wk<br>- DM 4(5)<br>- Current smokers 27(35), Ex-smoker 3(4)<br>- MTX cumulative dose; median 2.6(range 0.13-19.1) g, mean 4.1 g<br>- MTX duration; median 2.8(range 0.3-20.6) years, mean 5.1 years
Investigations	- TE, FibroTest (Fibrosure), serial PIIINP (≥ 3 abnormal results in 1 year)<br>- Liver biopsy
Outcomes	- TE < 7 kPa = 41(82)<br>- FibroTest < 0.31 = 59(84.3)<br>- Serial PIIINP (1 year before TE) > 4.2 = 9/51 (17.6)<br>- Serial PIIINP (1 year before FibroTest) > 4.2 = 3/34 (8.8)<br>- On univariate analysis body mass index (r = 0.40, P = .005) and age (r = 0.52, P = .005) were correlated with abnormal TE results.<br>- Age (r = 0.31, P = .009), cumulative methotrexate dose (r = 0.31, P = .01), and duration of methotrexate therapy (r = 0.36, P = .002) were correlated with abnormal FibroTest results.<br>- Steatosis was demonstrated in all 5 patients who received liver biopsies during the study. Two patients had hepatic fibrosis, with 1 showing a sinusoidal pattern of fibrosis attributed to steatohepatitis.
Comments	

Bray, 2012 (49)	
Methods	- Cross-sectional<br>- United Kingdom
Participants	- 21 psoriasis patients taking MTX (10 had valid TE scans, 7 failed to obtain a valid result)<br>- Age 59 (range 41-83)<br>- Female 9(42.9)<br>- Psoriasis type; chronic plaque 19(90), pustular 1(5), palmoplantar hyperkeratotic 1(5), psoriatic arthritis 7(33)<br>- MTX cumulative dose 4.927 (5.420 range 1.250-18.695) g<br>- BMI 33 kg/m^2
Investigations	- TE
Outcomes	- TE < 8 kPa = 6(60)<br>- TE ≥ 10 kPa = 3(30)<br>- TE ≥ 7.1 kPa = sensitivity 100% (95CI:15.8–100%), specificity 67% (95CI: 29.9–92.5%).
Comments	

Madanagobalane, 2012 (50)	
Methods	- Cross-sectional study<br>- India
Participants	- 58 psoriasis patients<br>- Age 46.9±1.15<br>- Female 12(20.7)<br>- BMI 32.3±5.2<br>- PASI score 6.5±10.8<br>- Psoriatic arthritis 21 (36.2)<br>- Current smoker 13 (22.4)<br>- Obesity 54 (80)<br>- Metabolic syndrome 49 (84.48)<br>- Abdominal obesity 45 (77.5), 103.4±8.5<br>- Hypercholesterolemia 41 (69.4)<br>- Hypertriglyceridemia 48 (82.7)<br>- Hyperglycemia 42 (72.4)<br>- Hypertension 20 (34.4)<br>- Diabetes 26 (44.8)<br>- Patients on current treatment and those who received cyclosporine, acitretin, psoralens and methotrexate within the last 6 weeks were excluded from the study.
Investigations	- FibroTest
Outcomes	- FibroTest stage < F2-F3 = 94.9%
Comments	

Seitz, 2010 (51)		
Methods	- Prospective, cross-sectional study<br>- Switzerland	
Investigations	- TE	
Participants	TNF- (N=20)<br>- Age 51.9±14.1 years<br>- Female 6(30.0)<br>- BMI 26.4±3.8 kg/m^2<br>- Psoriasis duration 9.5±7.6 years<br>- MTX use 100%<br>- MTX dose/wk 16.1±4.6 mg<br>- MTX cumulative 2.7±2.3 g<br>- Folic cumulative 9.8±5.0 g<br>- Diabetes 3(15.0)<br>- Steatohepatitis 4(20.0)<br>- Dyslipidemia 4(20.0)<br>- Alcohol use 7(35.0) %<br>- Alcohol use 11.3±16.6 g/day<br>- Abnormal LFT 7(35.0)	TNF+ (N=23)<br>- Age 51.3±10.9<br>- Female 7(30.4)<br>- BMI 27.0±4.5<br>- Psoriasis duration 11.8±11.2 years<br>- MTX use 100%<br>- MTX dose/wk 15.4±5.4 mg<br>- MTX cumulative 3.7±3.2 g<br>- Folic cumulative 9.4±3.0 g<br>- Diabetes 3(13.0)<br>- Steatohepatitis 3(13.0)<br>- Dyslipidemia 3(13.0)<br>- Alcohol use 5(21.7) %<br>- Alcohol use 4.3±10.4 g/day<br>- Abnormal LFT 10(43.5)
Outcomes	- TE < 8 kPa = 14(70)	- TE < 8 kPa = 22(95.7)
Comments		

Laharie, 2010 (52)	
Methods	- Prospective, case-control study<br>- France
Participants	- 111 psoriasis patients<br>- Female 30(27)<br>- Age 56.2±12.2<br>- BMI 27±4.8 kg/m^2<br>- Diabetes 15(13.5)<br>- Hypertension 34(30.6)<br>- Alcohol use 3(0-16.2)<br>- MetS 25(25.8)<br>- MTX naive 28(25.2)<br>- MTX cumulative dose 1.56(0.48-3) g<br>- MTX dose/wk 110(32-250) mg, 0.110(0.032-0.250) mg<br>- AST 33(22-47) IU/L<br>- GGT 36(21-72) IU/L
Investigations	- TE, liver biopsy
Outcomes	- TE <7.9 kPa = 99(89.2)<br>- 6 of 12 was biopsied
Comments	

Lindsay, 2009 (56)	
Methods	- Prospective study<br>- United Kingdom
Participants	- 54 patients with psoriasis (48 with PIIINP results)<br>- Mean age 54.4±11 years<br>- Psoriatic arthritis 47/54(87%)<br>- Psoriasis duration 27.3±15 years<br>- Arthritis duration 18.6 ± 13 years<br>- Mean MTX treatment duration 6.9±4.22 years<br>- Mean MTX weekly dose of 0.0155 ± 0.00617 g, 15.5 ± 6.17 mg<br>- Mean MTX cumulative dose of 4.396±3.140 g, 4396±3140 mg
Investigations	- PIIINP<br>- Liver biopsy
Outcomes	- elevated PIIINP = 16 (33.3)<br>- no cases of advanced fibrosis or of cirrhosis and mild early fibrosis in 11 (22%) patients<br>- 4 of those with early fibrosis had a normal PIIINP on the day of their liver biopsy; in seven patients it was elevated
Comments	

Berends, 2007 (66)	
Methods	- Netherlands
Participants	- 24 psoriasis patients who had a recent liver biopsy during MTX use<br>- Female 13 (54.2)<br>- Age 55 (range 34–73) years<br>- BMI 26 (20–38) kg/m^2<br>- Diabetes 4 (17)<br>- MTX cumulative dose 3.352 (0.314–20.235) g<br>- MTX cumulative dose > 5g 4(16.7)
Investigations	- Fibroscan<br>- FibroTest<br>- Liver biopsy
Outcomes	- TE >7.1 kPa [Metavir ≥F2] = sensitivity 50%, specificity 88%, accuracy 70%, PPV 33%, NPV 86%<br>- FibroTest >0.31 [Metavir ≥F2] = sensitivity 83%, specificity 61%, accuracy 67%, PPV 42%, NPV 92%<br>- Fibroscan values (n=20) ranged between 3.3 and 18.4 kPa (median value 6.4 kPa) and correctly identified 88% of the patients without significant liver fibrosis (Metavir score < F2, Fibroscans ≤ 7.1 kPa).<br>- The Fibrotest identified 83% of the patients with significant liver fibrosis (Metavir score <F2, Fibrotest ≥0.31)
Comments	

Khan, 2006 (64)	
Methods	- Retrospective<br>- United Kingdom
Participants	- 65 patients with moderate to severe psoriasis receiving MTX<br>- 15 patients had a total of 30 biopsies (Liver biopsies were performed according to guidelines with three abnormal (>4.2 ug/l) results in 12 months.<br>- Age 56.4 ± 12.8, female 7 (46.7%)<br>- Total number of MTX years was 278.9 with a follow up period of 1–14 years with a mean duration of 4.3 (SD 3.9) years.<br>- Mean cumulative dose of MTX 2.000 (SD 1.838) g
Investigations	- PIIINP
Outcomes	- 174 P3NP assays and 30 liver biopsies were recorded.<br>- 16 P3NP estimations (28%)—that is, value >4.2 ug/l of a total of 58 correlated at some stage with an abnormal liver biopsy.<br>- Most (11, 73.3%) of them had either normal biopsy findings or mild to moderate steatosis
Comments	

Zachariae, 2001 (57)	
Methods	- Retrospective study<br>- Denmark
Participants	- 70 patients with psoriasis, who were on MTX and had both a liver biopsy without fibrosis and a normal PIIINP<br>- The follow-up time was from 1 to 11 years.
Investigations	- PIIINP<br>- Liver biopsy
Outcomes	- PIIINP > 4.2 μg/L = 6(8.6)<br>- A total of 189 liver biopsies and 329 analyses of PIIINP were recorded.<br>- 21 patients had only one and no further biopsies, but their data included at least two to three PIIINP samples obtained within a year around the time of the biopsy, and at least two were taken either prior to or at the time of the biopsy.<br>- 49 patients had from 2 to 7 liver biopsies each and a total of 267 analyses of PIIINP.<br>- 4 patients developed fibrosis of the liver as shown by liver biopsies, and all of these patients developed elevated serum PIIINP levels.<br>- 2 further patients had elevated PIIINP, but normal liver biopsy.<br>- Thus, no liver fibrosis was missed in the 63 patients with consistently normal PIIINP levels.
Comments	

Zachariae, 1996 (59)	
Methods	- Denmark
Participants	- The investigations were carried out on 186 liver biopsies and 5 autopsies.<br>- 11 surviving psoriasis patients were also studied by analysis of serum aminoterminal propeptide of type III procollagen (PIIINP),
Investigations	- PIIINP<br>- Liver biopsy
Outcomes	- PIIINP > 4.2 μg/L = 0(0)
Comments	

Boffa, 1996 (60)	
Methods	- United Kingdom
Participants	- 87 patients receiving long-term MTX treatment for severe psoriasis.
Investigations	- PIIINP
Outcomes	- PIIlNP-O > 4.2 µg/L = 41(47.1)<br>- PIIlNP-O Sensitivity 81%, specificity 62%
Comments	

Oogarah, 1995 (63)	
Methods	- United Kingdom
Participants	- 22 psoriasis patients<br>- Female 8 (36.4%)<br>- Mean age 42.6 (22-72) years<br>- Duration of treatment 188.5(35-738) weeks<br>- The total cumulative dose of MTX 1.901 (0.395-5.645) g
Investigations	- PIIINP<br>- Liver biopsy
Outcomes	- Elevated P3NP levels were found in 6 of 11 (54.5) patients with the first assay ($P3NP_1$)<br>- Elevated P3NP levels were found in 4 of 22 (18.2) patients with the second assay (P3NP2)<br>- Only two patients had raised levels with both assays.
Comments	- 11 of the patients were included in Mitchell 1990, and thus was not included in the quantitative analysis

Zachariae, 1991 (58)	
Methods	– Denmark
Participants	- 170 patients with psoriasis; 83 (48.8%) had PsA<br>- who had liver biopsies performed during or before treatment with methotrexate or, in some cases, with retinoids.
Investigations	- PIIINP<br>- Liver biopsy
Outcomes	- Psoriasis patients with fibrosis or cirrhosis in their liver biopsy specimens had a significantly higher mean serum P3NP than did patients without fibrosis and without arthritis.<br>- PIIINP > 4.3 µg/L = 24(21.8); 2 of 52 (3.85%) of patients without cirrhosis or fibrosis and no arthritis had an elevated P3NP, 22 of 58 (37.93%) of patients with PsA had an increased P3NP in the absence of detectable liver fibrosis.
Comments	

Mitchell, 1990 (61)	
Methods	- United Kingdom
Participants	- 51 patients with severe psoriasis<br>- Age 47 (range 22-69) years<br>- Duration of MTX treatment 5.2 (1-13) years<br>- Oral MTX cumulative dose 2.538 (0.545-10.024) g<br>- control group; 18 healthy patients with non-inflammatory skin disorders, and 18 active psoriasis patients who had never received systemic treatment for psoriasis
Investigations	- PIIINP (referece range 2.0-11.8 μg/L)
Outcomes	- PIIINP > 11.8 μg/L = 35(68.6)<br>- The normal reference range for serum levels of P3NP quoted by the assay manufacturers is 4-12 ng/ml. None of the subjects in the normal or psoriatic control groups had elevated serum P3NP levels (range 2.0-11.8 μg/L)
Comments	

Risteli, 1988 (62)	
Methods	- Denmark
Participants	- 24 patients with psoriasis and on methotrexate<br>- Female 10 (41.7)<br>- Age 50 (32-75) years
Investigations	- PIIINP<br>- Liver biopsy
Outcomes	- PIIINP > 4.2 µg/L = 8(33.3); 8 of 9 patients had abnormal PIIINP level, and all 9 had abnormal liver biopsy. 15 patients that had normal level of PIIINP; 9 had normal liver biopsies, and 2 had minimal liver fibrosis.
Comments	
