# Supplementary material for: Liver fibrosis prevalence and risk factors in patients with psoriasis: A systematic review and meta-analysis
Source: Front Med (Lausanne). 2022 Dec 15;9:1068157. doi: 10.3389/fmed.2022.1068157 (PMC9797863; doi:10.3389/fmed.2022.1068157)
Supplement: Supplementary file 4 [file Table_3.pdf]

Supplementary Table 3: Formulae of non-invasive tests

**Fibrosis-4 (FIB-4) index**	(Age[years] × AST[U/L]) / (platelet count [x10^9/L] × √ALT[U/L])
**AST to Platelet Ratio Index (APRI)**	[(AST/upper limit of the normal AST range) × 100]/platelet count [x10^9/L]
**NAFLD fibrosis score (NFS)**	-1.675 + 0.037 × age (years) + 0.094 × BMI (kg/m2) + 1.13 × diabetes (yes = 1, no = 0) + 0.99 × AST/ALT ratio - 0.013 × platelet count (x10^9/L) - 0.66 × albumin (g/dL)
**FibroSure/ FibroTest**	4467 × log [alpha2-macroglobulin (gr/l)] – 1357 × log [haptoglobin (gr/l)] + 0.0821 × [age (years)] +1737 × log [bilirubin (μmol/l)] - 1184 × [apolipoprotein A1 (gr/l)] + 0.301 × sex (male=1, female=0) – 5.054
**Enhanced Liver Fibrosis score (ELF)**	2.494+0.846ln(CHA)+0.735ln(CPIIINP)+0.391ln(CTIMP−1); amino-terminal propeptide of type III procollagen (PIIINP), tissue inhibitor of matrix metalloproteinase 1 (TIMP-1) and hyaluronic acid (HA)
**Forns Index**	7.811 - 3.131 x ln(platelet count) + 0.781 x ln(GGT) + 3.467 x ln(age) - 0.014 x (cholesterol)
**FibroMeter NAFLD**	0.4184 glucose (mmol/l) + 0.0701 AST (IU/l) + 0.0008 ferritin (μg/l) - 0.0102 platelet (G/l) - 0.0260 ALT (UI/l) + 0.0459 body weight (kg) + 0.0842 age (yr) + 11.6226.
**Hepascore**	y/(1 + y), where y = exp (-4.185818 – 0.0249 × age(years) + 0.7464 ×gender (male = 1, female = 0) + 1.0039 × α2-macroglobulin + 0.0302 ×hyaluronic acid + 0.0691 × bilirubin
**Fibrosis Probability Index (FPI)**	10.929 + (1.827 × Ln AST) +(0.081 × age) + (0.768 × past alcohol use) + (0.385 × HOMA-IR) – (0.447 × cholesterol)
