# Supplementary material for: Liver fibrosis prevalence and risk factors in patients with psoriasis: A systematic review and meta-analysis
Source: Front Med (Lausanne). 2022 Dec 15;9:1068157. doi: 10.3389/fmed.2022.1068157 (PMC9797863; doi:10.3389/fmed.2022.1068157)
Supplement: Supplementary file 5 [file Table_4.pdf]

Supplementary Table 4: Summary of meta-regression analysis for pooled prevalence of high risk and low risk of advanced liver fibrosis, respectively.

**Variables**	**β**	**SE**	**z**	**p-value**	**95%CI**	
					**LCI**	**UCI**
N	0.01122	0.02369	0.47	0.636	-0.03520	0.05765
Age	0.11734	0.43259	0.27	0.786	-0.73052	0.96521
Female proportion	-0.04602	0.2096	-0.22	0.826	-0.45683	0.36480
BMI	1.53033	1.1183	1.37	0.171	-0.66150	3.72216
Obesity proportion	0.29904	0.17633	1.70	0.090	-0.04655	0.64463
Psoriasis duration	0.20335	0.47688	0.43	0.670	-0.73132	1.13802
PASI	-0.3461	0.68818	-0.50	0.615	-1.69491	1.00270
MTX user proportion	0.0004	0.0006	0.66	0.507	-0.07877	0.15831
DM proportion	-0.04645	0.25981	-0.18	0.858	-0.55567	0.46278
HT proportion	-0.00019	0.14857	-0.00	0.999	-0.29138	0.29101
DLP proportion	0.0794	0.17499	0.45	0.650	-0.26356	0.42237

**Variables**	**β**	**SE**	**z**	**p-value**	**95%CI**	
					**LCI**	**UCI**
N	0.00566	0.01874	0.30	0.763	-0.03107	0.04238
Age	-0.32552	0.31215	-1.04	0.297	-0.93733	0.28628
Female proportion	-0.14755	0.18902	-0.78	0.435	-0.51802	0.22292
BMI	-0.19998	0.43024	-0.46	0.642	-1.04324	0.64328
Obesity proportion	-0.13019	0.12437	-1.05	0.295	-0.37395	0.11358
Psoriasis duration	-0.86971	0.4862	-1.79	0.074	-1.82264	0.08323
PASI	-0.86466	0.60412	-1.43	0.152	-2.04870	0.31938
PsA proportion	-0.10206	0.20558	-0.50	0.620	-0.50500	0.30088
MTX user proportion	-0.07245	0.05685	-1.27	0.203	-0.18388	0.03898
MTX cumulative dose	-2.95996	3.11586	-0.95	0.342	-9.06693	3.14702
DM proportion	-0.00509	0.25306	-0.02	0.984	-0.50108	0.49089
DLP proportion	0.15152	0.14602	1.04	0.299	-0.13468	0.43772
MetS proportion	0.07454	0.36615	0.20	0.839	-0.64311	0.79219
