# Supplementary material for: Liver fibrosis prevalence and risk factors in patients with psoriasis: A systematic review and meta-analysis
Source: Front Med (Lausanne). 2022 Dec 15;9:1068157. doi: 10.3389/fmed.2022.1068157 (PMC9797863; doi:10.3389/fmed.2022.1068157)
Supplement: Supplementary file 6 [file Table_5.pdf]

Supplementary Table 5: The quality assessment of studies included in the systematic review

First author, year	Selection				Comparability of the study population (0-2)	Outcome assessment (0-1)	Statistical test (0-1)	Total score (0-9)
	Representativeness (0-1)	Sample size (0-1)	Fibrosis as outcome of interest (0-1)	Fibrosis assessment tool (0-2)				
Lee, 2022 (27)	1	1	1	2	2	1	1	9
Mahajan, 2022 (30)	1	1	1	2	2	1	1	9
Takamura, 2022 (28)	1	1	1	2	2	1	1	9
Rattanakaemakorn, 2021 (31)	1	1	1	2	2	1	1	9
Belinchón-Romero, 2021 (32)	1	1	1	2	2	1	1	9
Brunner, 2021 (33)	1	1	1	2	2	1	1	9
Cervoni, 2020 (34)	1	1	1	2	2	1	1	9
Yim, 2020 (19)	1	1	1	2	1	1	1	8
Rivera2020 , (35)	1	1	1	2	2	1	1	9
Koch, 2020 (36)	1	1	1	2	2	1	1	9
Mahajan, 2020 (29)	1	1	1	2	2	1	1	9
Magdaleno-Tapial, 2020 (37)	1	1	1	2	2	1	1	9
Kumar, 2020 (38)	1	1	1	2	1	1	1	8

First author, year	Selection				Comparability of the study population (0-2)	Outcome assessment (0-1)	Statistical test (0-1)	Total score (0-9)
	Representativeness (0-1)	Sample size (0-1)	Fibrosis as outcome of interest (0-1)	Fibrosis assessment tool (0-2)				
Neema, 2020 (39)	1	1	1	2	2	1	1	9
Ortolan, 2019 (40)	1	1	1	2	2	1	1	9
BenLagha, 2019 (41)	1	1	1	2	2	1	1	9
Maybury, 2019 (42)	1	1	1	2	2	1	1	9
vandenReek, 2017 (55)	1	1	1	1	1	1	1	7
vanderVoort, 2017 (54)	1	1	1	2	2	1	1	9
Bauer, 2017 (43)	1	1	1	2	2	1	1	9
Talme, 2017 (44)	1	1	1	2	2	1	1	9
Rongngern, 2017 (45)	1	1	1	2	2	1	1	9
Pongpit, 2016 (46)	1	1	1	2	2	1	1	9
Gisondi, 2016 (47)	1	1	1	2	2	1	1	9
vanderVoort, 2016 (53)	1	1	1	2	2	1	1	9
Martyn-Simmons, 2014 (65)	1	1	1	2	1	1	1	8
Lynch, 2014 (48)	1	1	1	2	2	1	1	9

First author, year	Selection				Comparability of the study population (0-2)	Outcome assessment (0-1)	Statistical test (0-1)	Total score (0-9)
	Representa tiveness (0-1)	Sample size (0-1)	Fibrosis as outcome of interest (0-1)	Fibrosis assessment tool (0-2)				
Bray, 2012 (49)	1	0	1	2	2	1	1	8
Madanagobalane, 2012 (50)	1	1	1	2	2	1	1	9
Seitz, 2010 (51)	1	1	1	2	2	1	1	9
Laharie, 2010 (52)	1	1	1	2	2	1	1	9
Lindsay, 2009 (56)	1	1	1	1	1	1	1	7
Berends, 2007 (66)	1	0	1	2	2	1	1	8
Khan, 2006 (64)	1	1	1	1	0	1	0	5
Zachariae, 2001 (57)	1	1	1	1	0	1	0	5
Zachariae, 1996 (59)	1	0	1	1	0	1	0	4
Boffa, 1996 (60)	1	1	1	1	0	1	1	6
Oogarah, 1995 (63)	1	0	1	1	1	1	0	5
Zachariae, 1991 (58)	1	1	1	1	0	1	1	6
Mitchell, 1990 (61)	1	1	1	1	1	1	1	7
Risteli1988 , (62)	1	0	1	1	1	1	0	5
